# Directed transforming of coke to active intermediates in methanol-to-olefins catalyst to boost light olefins selectivity

**DOI:** 10.1038/s41467-020-20193-1

**Published:** 2021-01-04

**Authors:** Jibin Zhou, Mingbin Gao, Jinling Zhang, Wenjuan Liu, Tao Zhang, Hua Li, Zhaochao Xu, Mao Ye, Zhongmin Liu

**Affiliations:** 1 National Engineering Laboratory for Methanol to Olefins, Dalian National Laboratory for Clean Energy, Dalian Institute of Chemical Physics, Chinese Academy of Sciences, Dalian, China; 2 University of Chinese Academy of Sciences, Beijing, China; 3 Key Laboratory of Separation Science for Analytical Chemistry, Dalian Institute of Chemical Physics, Chinese Academy of Sciences, Dalian, China

**Keywords:** Catalytic mechanisms, Heterogeneous catalysis, Chemical engineering

## Abstract

Methanol-to-olefins (MTO), the most important catalytic process producing ethylene and propylene from non-oil feedstocks (coal, natural gas, biomass, CO_2_, etc.), is hindered by rapid catalyst deactivation due to coke deposition. Common practice to recover catalyst activity, i.e. removing coke via air combustion or steam gasification, unavoidably eliminates the active hydrocarbon pool species (HCPs) favoring light olefins formation. Density functional theory calculations and structured illumination microscopy reveal that naphthalenic cations, active HCPs enhancing ethylene production, are highly stable within SAPO-34 zeolites at high temperature. Here, we demonstrate a strategy of directly transforming coke to naphthalenic species in SAPO-34 zeolites via steam cracking. Fluidized bed reactor-regenerator pilot experiments show that an unexpectedly high light olefins selectivity of 85% is achieved in MTO reaction with 88% valuable CO and H_2_ and negligible CO_2_ as byproducts from regeneration under industrial-alike continuous operations. This strategy significantly boosts the economics and sustainability of MTO process.

## Abstract (web summary)

Methanol-to-olefins is hindered by rapid catalyst deactivation due to coke deposition. Here the authors demonstrate an approach of directly transforming coke to active intermediates to simultaneously recover catalyst activity and boost light olefins selectivity.

## Introduction

Since its first commercialization in 2010^1^, methanol-to-olefins (MTO) over zeolitic catalyst has become the most important process producing light olefins (ethylene and propylene) from non-oil feedstocks such as coal, natural gas, biomass, and CO_2_^1–5^. Despite essential progress achieved in both fundamental and applied research in the past decades, concurrently pursuing long catalyst lifetime and high light olefins selectivity in MTO remains an open challenge so far^2,6^. As an archetypical co-catalysis reaction, catalytic performance in MTO is manipulated by the organic intermediates confined in zeolite channels or cavities through the sophisticated hydrocarbon pool mechanism^3,7–9^. These hydrocarbon pool species (HCPs), typically including the methyled-benzene carbocations^10,11^ and cyclopentadienyl species^12,13^, are decisive for light olefins selectivity, owning to the altering of acidity^14^, reaction paths^15^, kinetics^8,9^, molecular transport^16^, and among others. However, the HCPs are also coke precursors that can readily evolve to polycyclic aromatic hydrocarbons (PAHs), the typical coke species, through cyclization^17^ and cross-linked mechanism^18,19^, accelerating catalyst deactivation^20^. Therefore, such dual-role of HCPs impedes achieving superior light olefins selectivity while maintaining long catalyst lifetime in MTO over zeolitic catalyst. In catalytic processes accompanying with catalyst deactivation by coke deposition, e.g., MTO and fluid catalytic cracking (FCC), air combustion or steam gasification^21,22^ has been used as common practices to eliminate coke for catalytic activity recovering. This unavoidably lowers light olefins selectivity because the active HCPs favoring light olefins formation in the catalyst have been meantime eliminated. However, the reinstitution of HCPs in nano-cavity of zeolite catalyst is of equal importance in recovering acidity and prompting light olefins selectivity. In this connection, a logical question follows: can the coke inevitably causing catalyst deactivation be selectively transformed to active HCPs?

Some methods have been proposed to modulate the product selectivity via tailoring the HCPs confined in zeolites for MTO reactions. By use of mimics of the HCPs as organic structure-directing agents (OSDAs) to synthesize CHA and RTH-related zeolites, it is possible to maximize the host–guest interaction between framework and polymethyl aromatic intermediates to promote the propylene/ethylene ratio^8,11^. However, precise design and synthesis of targeted zeolites with specific OSDAs are extremely challenging^23^, especially the HCPs slightly different in structures may significantly change the product selectivity^8^. Co-feeding of methanol with organic intermediates, e.g., alkenes^24^, aromatics^25^ or formaldehyde^26^, has also been applied to adjust ethylene/propylene ratio via altering the HCPs dominating either olefinic or aromatic cycle^2,6^ according to dual-cycle mechanism. Unfortunately, co-feeding of aromatics suffers significant diffusion limitation owning to the steric hindrance of nanopore of zeolite framework^1,27^. Pre-synthesis specific active HCPs inside vacant cavity of SAPO-34 zeolite by methanol^28^ or n-butene^15^ at high temperatures can also favor ethylene formation. In the aforementioned methods, nevertheless, the light olefins selectivity can be modulated to certain extent by tuning HCPs with assistance of external reactants. Formation of coke and thus catalyst deactivation, as a result of evolution of HCPs within zeolites, are still major concern in these methods.

**Fig. 1:**
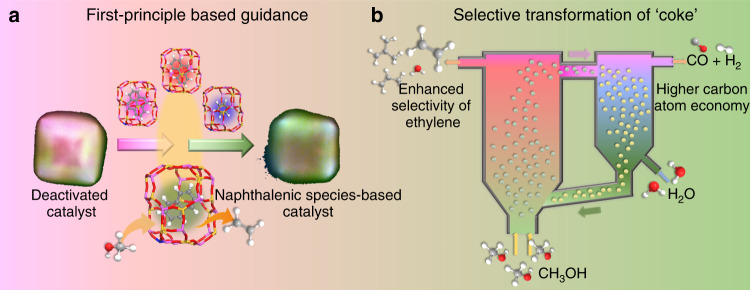
Schematic illustrations of selective transformation of coke into specific intermediates. **a** First-principle-based simulations provide the criteria of stability and functionality of organic intermediates confined in nano-cavity. **b** Selective transformation of coke into specific naphthalenic species-rich catalyst, and improvement of MTO performance and atom economy implemented in the circulating fluidized bed reactor-regenerator configuration.

Here, we demonstrate a regeneration strategy to directionally transform coke to active HCPs within nano-confined spaces of industrially important SAPO-34 zeolites. Theoretically the steric stability and functionality are two metrics for screening scaffold of HCPs confined in nano-space^17^. We first conduct density functional theory (DFT) calculations and find that naphthalenic species, which favor ethylene formation are spatially-stable within CHA cavity at high temperature. Thus coke within the deactivated catalyst, i.e., mainly PAHs, can in principle be transformed to naphthalenic species via thermal cracking at high temperature. In doing so, we sweep coked SAPO-34 catalyst with nitrogen under high temperature and obtain naphthalenic species-rich SAPO-34 zeolite (Fig. 1a), which exhibits high light olefins selectivity but poor methanol conversion. We further study two mediums commonly used for ring-opening of PAHs, i.e., hydrogen and steam, via DFT calculations. Comparison of energy barriers shows that steam, despite being the main byproduct in MTO, is more effective in cracking coke to naphthalenic species in SAPO-34 zeolites. Laboratory experimental results demonstrate that directionally transforming coke confined in SAPO-34 zeolites to naphthalenic species by steam cracking, not only restores the catalytic activity but also promotes light olefins selectivity in MTO reaction. In addition, steam cracking of coke releases only a small amount of byproducts in the form of flue gas, which is dominantly composed of valuable syngas (H_2_ and CO) with negligible greenhouse gas CO_2_ (Fig. 1b). By verifying it in a fluidized bed reactor-regenerator pilot plant, we show this strategy can significantly boost the economics and sustainability of industrial MTO process.

## Results

### Stability and functionality of intermediates confined in CHA cavity

**Fig. 2:**
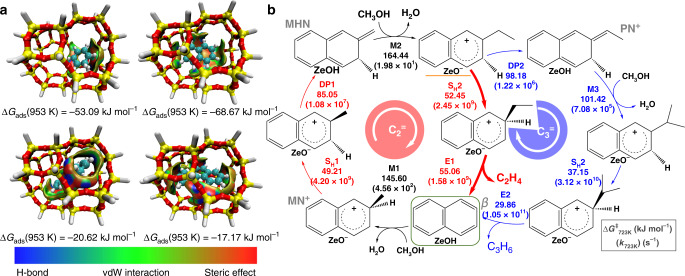
Guidance from density functional theory to screen the stable and functional intermediates. **a** Isosurface plots of the reduced density gradient and Gibbs free energy of adsorption at 953 K for the benzenic, naphthalenic, phenanthrenic, and pyrenic carbenium ions confined in neutral pure silica model of CHA cavity. The isosurfaces of the reduced density gradient are colored according to the values of the quantity sign(*λ*_2_)*ρ* with the indicated RGB scale. vdW represents the van der Waals interactions. **b** Proposed side-chain mechanism in SAPO-34 zeolite for ethylene (C_2_^=^) and propylene (C_3_^=^) formation starting from sterically stabilized naphthalene. Gibbs free energy in kJ mol^−1^ and intrinsic rate coefficient in 1 s^−1^ (in parentheses) at 723 K.

It is well recognized that the organic intermediates confined in nano-cavity play the crucial role in determining the product selectivity for MTO reaction^7,9–11^. As reaction proceeds, the transformation of monocyclic aromatic to PAHs is inevitable^1^. Therefore, it is hard to solely identify the functionality of specific intermediates confined in zeolites during reaction by experiments. The steric stabilities of benzenic (B_n_^+^), naphthalenic (N_n_^+^), phenanthrenic (PH_n_^+^), and pyrenic (PYR_n_^+^) carbocations with *n* methyl substituent confined within negative SAPO-34 zeolite model (Supplementary Table 6) and neutral pure silica model of CHA cavity (Supplementary Table 7) at a high temperature (953 K), in terms of the adsorption Gibbs free energy (Δ*G*_ads_), are calculated by DFT. According to the calculated results, naphthalenic carbocation is the preferentially stable carbenium ions within negative SAPO-34 zeolite model and pure silica model of CHA at 953 K. In addition, this implies the dominated stabilization effect is imposed by CHA confinement. Similarly, the calculated results of neutral carbonaceous species within neutral SAPO-34 model containing Brønsted acid site, also verify that the preferentially stable carbonaceous species within CHA cavity is naphthalene species (Supplementary Table 8). In Fig. 2a, in the pure silica model of CHA, the isosurface plots of reduced density gradient for phenanthrenic and pyrenic carbocations exhibit enriched van der Waals (vdW) stabilization interaction at the border of molecules from the zeolite framework instead of enveloping the whole molecules like benzenic and naphthalenic carbocations. Moreover, owning to the small spatial occupancy of benzenic carbocations, the interaction imposed by zeolite framework is relatively weaker than that of naphthalenic carbocations. These confirm again that the chemical environment of CHA cavity leads to the more preferential stabilization of naphthalenic carbocations from the perspective of molecular level.

In Fig. 2b, the full catalytic cycle with Gibbs free energy and intrinsic rate coefficient at 723 K for ethylene and propylene formation starting from naphthalene is presented. To verify the calculations, the calculated results were compared with the previous work. The calculated Gibbs free energies of M1 and S_H_1 are 144.07 and 49.26 kJ mol^−1^ at 670 K, respectively, which are close to the calculated results by Hemelsoet et al.^29,30^ (136.4 and 36.4 kJ mol^−1^, respectively). The slightly difference in free energies may be caused by the different calculated approaches. The Gibbs free energy of M2 of 2-methylene-3-hydronaphthalene (MHN) is 161.68 kJ mol^−1^ at 670 K, which is lower than exocyclic methylation of the 1,1-methy-2-methylene-hydronaphthalene at 670 K (175.7 kJ mol^−1^)^30^. Owing to the strong steric hindrance imposed by two methyl of 1,1-dimethy-2-methylene-hydronaphthalene during exocyclic methylation, the Gibbs free energy of M2 is lower than that of exocyclic methylation of 1,1-methy-2-methylene-hydronaphthalene. At 723 K, naphthalene undergoes methylation M1 at the position of the *β*-carbon atom (Fig. 2b), which shows high free energy barriers (145.60 kJ mol^−1^). However, at 723 K, the intrinsic rate coefficient can be effectively improved to 4.56 × 10^2^ s^−1^ compared to 0.16 × 10^2^ s^−1^ of 623 K (Supplementary Table 10), which means the reaction is more feasible through elevating temperature and naphthalene species can be considered as active HCPs at 723 K. Subsequently, 2-methyl-naphthalenic cation (MN^+^) gets hydride-shifted (S_H_1) and deprotonated (DP1), with the formation of (MHN) with moderate free energy barriers of 49.21 and 85.05 kJ mol^−1^, respectively. In the following step, the exocyclic double bond of MHN gets methylated (M2) and the exocyclic methylation M2 is strongly exothermic (Δ*H*_r_ = −81.72 kJ mol^−1^, Supplementary Table 9) and manifests a higher free energy barriers (164.44 kJ mol^−1^) than M1. Thus, this is one of the slowest steps in the full catalytic cycle. To realize the catalytic cycle starting from naphthalenic cations, elevating reaction temperature is necessary. For instance, the intrinsic rate coefficient can be effectively improved from 0.07 × 10^1^ s^−1^ at 623 K to 1.98×10^1^ s^−1^ at 723 K for M2 step. In this work, the Gibbs free energies of formation of ethylene and propylene from naphthalene derivative were calculated. Then the exocyclic hydrogen migrates relatively fast (2.45 × 10^9^ s^−1^) toward the ring carbon whereto the ethyl side-chain is connected, which can be conducive to the broken of exocyclic C–C bonds for the ethylene split-off. The elimination E1 therewith promptly occurs through a concerted mechanism with rapid rate (1.58 × 10^9^ s^−1^) (Supplementary Table 10), in which the C–C bond is broken and the ethyl side-chain is deprotonated with ethylene formation simultaneously with the assistance of water^31,32^, and closes the catalytic cycle. The carbonaceous intermediates where the ethylene and propylene formation cycles bifurcate are indicated with orange line in Fig. 2b. The fashion of propylene formation is similar to that of ethylene, but the ethyl side-chain in the product of methylation M2 undergoes a second exocyclic deprotonation DP2 and methylation M3, with intrinsic rate coefficients three orders of magnitude lower compared to S_H_2 in Fig. 2b. This bifurcation (orange line in Fig. 2b) determines that the formation of ethylene is more preferential in catalytic cycle starting from naphthalenic cations. After rapid hydride-shift of hydro 2-propyl-3-hydronaphthalene (PN^+^), propylene is easily split off by elimination E2 in a concerted step. The role of naphthalenic cations in MTO reaction hereto has been well elucidated based on the DFT calculations. DFT calculations provide guidance that at the high reaction temperature (e.g., 723 K), the catalytic cycle starting from naphthalenic cations is more inclined to increase the selectivity of ethylene than propylene (Supplementary Table 9), to our best knowledge, which has not been reported so far.

### Transformation of coke to naphthalene in SAPO-34 zeolites

**Fig. 3:**
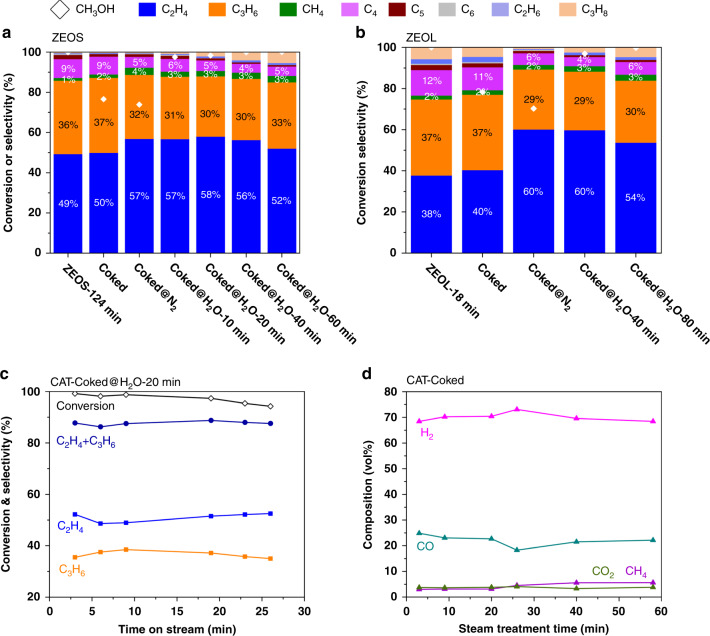
Catalytic performances over naphthalene-based SAPO-34 zeolites in MTO reaction. **a** ZEOS and **b** ZEOL zeolites were used to implement the strategy of selective transformation of coke to naphthalene. MTO reactions were first performed on calcinated SAPO-34 zeolites, the peak selectivity of ethylene and product distribution under completed conversion of methanol at 124 min for ZEOS sample (abbreviated as ZEOS-124 min) or at 18 min for ZEOL sample (abbreviated as ZEOL-18 min) are shown in the first bar chart. When the methanol conversion decreased to ~80%, the product distribution is shown in the second bar (samples named as ZEOS-Coked and ZEOL-Coked). The initial product distributions of MTO reaction at ~1.5 min are shown in the followed bar charts over ZEOS-Coked and ZEOL-Coked samples treated by nitrogen at 953 K for 40 min (samples aliased as Coked@N_2_) or steam cracking at 953 K for different times (samples aliased as Coked@H_2_O-xxmin series). MTO reaction condition: *T* = 723 K, weight hour space velocity (WHSV) = 6.6 g_MeOH_ g_cat._ h^−1^. Steam cracking condition: *T* = 953 K, WHSV = 2.6 g_Steam_ g_cat._ h^−1^. **c** MTO reaction performance over the CAT-Coked@H_2_O-20 min sample in the fluidized bed rector. MTO reaction condition: *T* = 723 K, WHSV = 2 g_MeOH_ g_cat._ h^−1^. Steam cracking condition: *T* = 953 K, WHSV = 3 g_Steam_ g_cat._ h^−1^. **d** Gas composition during steam cracking of CAT-Coked sample at *T* = 953 K and WHSV = 3 g_Steam_ g_cat._ h^−1^.

**Fig. 4:**
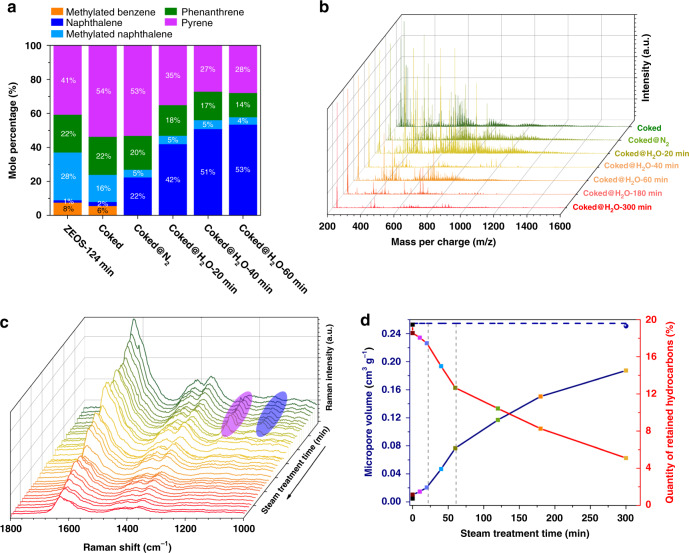
Evolution of carbonaceous species and textual property of ZEOS-Coked samples. **a** Evolution of detailed distribution of carbonaceous intermediates with molecular mass smaller than 200 Da during nitrogen and steam cracking at 953 K, which is analyzed by GC-MS. **b** Evolution of carbonaceous species with molecular mass larger than 200 Da during nitrogen and steam cracking at 953 K, which is analyzed by MALDI FT-ICR MS. **c** Operando UV-Raman spectra of ZEOS-Coked samples treated by steam at 923 K with different time. **d** Evolution of textual property and coke quantity of ZEOS-Coked samples during steam cracking at 953 K.

Two SAPO-34 zeolites with different crystal size, namely ZEOS (SAPO-34 zeolites with small average crystal size of ~1 μm) and ZEOL (SAPO-34 zeolites with large average crystal size of ~10 μm), are evaluated for MTO reaction in the fixed bed reactor. X-ray diffraction (XRD), scanning electron microscopy (SEM), and X-ray flurorescence (XRF) of these two samples are shown in Supplementary Figs. 1, 2 and Supplementary Table 2. Details of sample symbols and their preparation methods used in this work can be seen in Supplementary Table 1. According to DFT calculations (Fig. 2a), in CHA cavity, naphthalenic cations are the most electrostatic stabilization at high temperature and more inclined to favor ethylene formation (Fig. 2b). Moreover, the adsorption entropy exhibits different and negative values for different intermediates (Supplementary Tables 6–8). This implies the intrinsic difference of steric stability among intermediates can be further enlarged by elevating temperature. Intuitively, the simplest way to obtain the naphthalenic cations-based catalyst is to treat the coked catalyst at a higher temperature. Compared with the results of ZEOS-Coked (ZEOS sample treated by MTO reaction at 723 K for 175 min with methanol conversion of 80%), the proportion of naphthalene among the coke species with molecular mass smaller than 200 Da analyzed by gas chromatography-mass spectrometer (GC-MS) is remarkably enhanced from 2 to 22% for ZEOS-Coked@N_2_ (ZEOS-Coked sample treated by nitrogen at 953 K for 40 min) (see below-underlying mechanisms of selective transformation of coke). As shown in Supplementary Fig. 5, during the treatment by nitrogen through temperature programming, the coke amount begins slightly decreased when the temperature is above 773 K, but it tends to stabilize after a short sweeping period at 953 K. This confirms that coke deposited in the ZEOS-Coked@N_2_ sample has already stabilized. Then, the catalytic performances of these samples are evaluated. Figure 3a shows the results of ZEOS sample in MTO reaction at temperature of 723 K and time on stream (TOS) of 124 min with almost full methanol conversion (named as ZEOS-124 min hereafter), achieving a peak selectivity of ethylene of 49% (Supplementary Fig. 10). For ZEOS-Coked@N_2_ sample, the initial ethylene selectivity in MTO reaction at TOS of ~1.5 min significantly increases to 57% and the ethylene/propylene (*E/P*) ratio (~1.78) exceeds the maximum value (~1.36) achieved in MTO over fresh ZEOS sample. However, compared with the results with ZEOS-Coked sample, the methanol conversion (~75%) unfortunately shows no improvement. This may be related to the little recovery of pore volume and acidity in ZEOS-Coked@N_2_ sample (shown in Fig. 4d and Supplementary Fig. 17). Interestingly, the maximum *E/P* ratio in MTO reaction increases dramatically from 1.03 for ZEOL-18 min (ZEOL sample treated by MTO reaction at 723 K for 18 min, Supplementary Fig. 11) to 2.07 for ZEOL-Coked@N_2_ (ZEOL sample treated by MTO reaction at 723 K for 35 min and then treated by nitrogen at 953 K for 40 min), and the corresponding light olefins selectivity increases from 75% to 89% (Fig. 3b). These results are consistent with the DFT calculations, indicating that the presence of naphthalenic cations confined in CHA cavity makes the catalyst exhibits higher ethylene selectivity. However, it is practically desire to select a suitable medium to restore the catalytic conversion while keeping the dominance of naphthalenic species in the intermediates and maintaining the high ethylene selectivity.

Although air combustion can restore the catalytic activity, the introduction of aromatic C=O *sp*^2^ species, on the contrary, will eventually accelerate the catalyst deactivation^21,33^. Hydrogen may be a potential candidate^34,35^, however, the activation energy of hydrogenation reaction of PAHs (e.g., phenanthrene) with hydrogen is very high (220.47 kJ mol^−1^, Supplementary Fig. 7a and Supplementary Table 11), thus, the hydrogenation reaction normally occurs at the elevated pressure (ca. above 1.0 MPa)^34,35^. Steam, which is the major by-product in MTO reaction, can also act as H and OH radical donor to crack the C=C bond of aromatic rings with moderate activation energy with 110.11 and 102.43 kJ mol^−1^, respectively (Supplementary Fig. 7b and Supplementary Table 11). In additional, the high value-added H_2_ and CO are expected to produce during the splitting decomposition of hydroxyl-PAHs^36^. Therefore, steam is selected as the medium to restore the catalyst conversion.

Firstly, the effect of hydrothermal treatment on the physicochemical properties of ZEOS zeolite was studied. Results shown in Supplementary Figs. 3, 4 and Supplementary Tables 3–4 reveal that ZEOS with coke deposition has the improved hydrothermal stability (e.g., from the viewpoint of the acidity and micropore volume) than the fresh ZEOS after the same steam treatment condition (953 K for 300 min). As shown in Fig. 4d, with the increase of steam cracking time, the quantity of coke decreases with the gradual recovery of micropore volume and acidity (Supplementary Figs. 4 and 17). In Fig. 4a, the proportion of naphthalene also increases with the increase of treatment time for ZEOS-Coked@N_2_ samples treated by steam at 953 K (ZEOS-Coked@H_2_O). This suggests that, though the total amount of coke decreases during steam cracking, the predominance of naphthalene can be kept. In response to this, in Fig. 3a, b, it can be observed that not only the selectivity of ethylene can be held at the high level (~60%), but also the methanol can be completely converted for both ZEOS-Coked@H_2_O and ZEOL-Coked@H_2_O samples. Subsequently, a commercial SAPO-34 catalyst (termed as CAT, ~90 μm, catalyst pellet containing ZEOS, matrix and binder, Supplementary Fig. 2a) was evaluated in a micro-fluidized bed reactor at 723 K to examine the catalytic stability. A time independent product distribution can be observed in Fig. 3c, and the selectivity of light olefins can be maintained at around 88% within almost 25 min with nearly complete methanol conversion over CAT-Coked@H_2_O-20 min sample (CAT-Coked@N_2_ sample treated by steam at 953 K for 20 min). The selectivity of ethylene is almost maintained at 53%, corresponding to that achieved over fresh CAT sample during MTO reaction (Supplementary Fig. 8a), which successfully avoids the ascending stage of ethylene^23^. That is, MTO reaction can go straight into the “optimum operation window”^1^ by use of this coke transformation strategy. The influence of MTO reaction temperature on the catalytic performance was also investigated, and the results at 763 K are shown in Supplementary Fig. 9. As expected, the increase of MTO reaction temperature leads to a shortened catalytic lifetime and a higher *E/P* ratio, while the selectivity of light olefins still remains at 88% (Supplementary Fig. 9e). It needs to be emphasized that during steam cracking, the compositions of gas production are mainly H_2_ and CO, which can reach ~70 and ~20 vol%, respectively. Notably, this effectively improves the carbon atom economy than common air combustion strategy^1^.

### Underlying mechanisms of selective transformation of coke

Besides GC-MS, the matrix-assisted laser desorption/ionization Fourier-transform ion cyclotron resonance mass spectrometer (MALDI FT-ICR MS) was also used to obtain the complementary spectrum for the evolution of coke species^19^ ranged from 300 to 1600 Da confined in CHA cavity during steam cracking, as shown in Fig. 4b. The gradual decrease in the average molecular weight mass of coke species reveals that steam can act as the active agent in hydrogenation of the coke. This is also in agreement with the decreasing maximum temperature of differential curves (*T*_G,max_) obtained by thermogravimetric analysis (TGA) (Supplementary Fig. 5b), in which the *T*_G,max_ can reflect the nature of coke, i.e., the higher the *T*_G,max_ the lower the *H*/*C* ratio of coke^37^.

The vibrational fingerprints of coke species detected by operando UV-Raman characterization^38^ of ZEOS-Coked sample treated by steam at 923 K in the fixed bed reactor is shown in Fig. 4c. Normally the bands at 1630, 1415, and 1360 cm^−1^ are related to PAHs (e.g., naphthalene, phenanthrene, and pyrene)^39–42^, while the band at 1600 cm^−1^ corresponds to the intense G band of amorphous carbon^39^ and is overlapped with the band at 1630 cm^−1^. As can be seen, during steam cracking the intensities of peaks at 1630, 1415, and 1360 cm^−1^ decrease, indicating the elimination of PAHs. Meanwhile the appearance of the peak at 1600 cm^−1^ suggests the presence of amorphous carbon. And the morphology of amorphous carbon can be observed using field emission scanning electron microscopy (Supplementary Figs. 15, 16). This indicates that, although most of the coke undergoes hydrocracking during steam cracking, a part of coke is further condensed, which may follow the cross-lined mechanism^18^. Notably, with the evolution of the steam cracking time, the band at 1630 cm^−1^ slowly shifts to 1640 cm^−1^ and band at 1360 cm^−1^ to 1370 cm^−1^, which implies that there exist transformations from PAHs, e.g., phenanthrene (Raman shift at ~1365 cm^−1^) to naphthalene (Raman shift at ~1380 cm^−1^). Interestingly, the bands at 1240 and 1125 cm^−1^, which are identified as the exocyclic aromatic with branch chains of PAHs^41^, appear at the initial stage of steam cracking. This observation directly testifies that ring-open process of coke species is occurred during steam cracking.

### Underlying mechanisms of promotion of selectivity to ethylene

In addition to the theoretical results shown in Fig. 2, the ^12^C-/^13^C-methanol switch experiments over the ZEOS-Coked@H_2_O-40 min sample were performed to illustrate that naphthalenic species can serve as HCPs. As shown in Supplementary Figs. 12, 13a, for ZEOS-Coked@H_2_O-40 min sample, naphthalene is the main aromatic species while signals of methylated naphthalene species are very weak. After switching ^13^C-methanol at 723 K for 20, 40 and 60 s, the amount of naphthalene decreases promptly while the methylated naphthalene species increase significantly. Further MS analysis with the ^13^C label incorporation showed (Supplementary Fig. 13b) the methyl of methylated naphthalene is mainly derived from ^13^C-methanol. Likewise, the gas product, e.g., ethylene and propylene, are also mainly derived from ^13^C methanol (Supplementary Fig. 13c). These results obviously prove that naphthalenic species can act as HCPs to react with methanol during MTO reaction. As shown in Fig. 3a, compared to the ZEOS-124 min sample, ZEOS-Coked@H_2_O-40 min sample exhibits a higher ethylene selectivity, although these two samples have the nearly same coke amount (16.46 and 14.99 wt%, Supplementary Table 5). Checking the percentage of carbonaceous intermediates in these two samples depicted in Fig. 4a, the proportion of naphthalene and methylated naphthalene in ZEOS-Coked@H_2_O-40 min sample (51%) is significantly higher than that in ZEOS-124 min sample (30%). This implies that ethylene formation, to some degree, mainly depended on the naphthalenic species rather than the coke amount. In fact, the reactivity of naphthalenic species in MTO at high temperature within CHA zeolites was previously confirmed^10,43^.

Besides, to investigate the influence of naphthalenic species confined within CHA cavity on the molecular diffusion, the molecular dynamics (MD) simulations were conducted. The simulated intracrystalline diffusivities *D*_empty_ of ethylene and propylene in CHA zeolite at 723 K (two cavities contain one molecule) are 4.87 × 10^−10^ and 8.33 × 10^−12^ m^2^ s^−1^, respectively. When naphthalenic species loads within CHA cavity (two cavities contain one naphthalene molecule), the intracrystalline diffusivities *D*_load_ of ethylene and propylene decrease to 1.46 × 10^−10^ and 2.10 × 10^−12^ m^2^ s^−1^, respectively. It can be found that the ratio of *D*_empty_/*D*_load_ of ethylene is 3.34 and the ratio of *D*_empty_/*D*_load_ of propylene is 3.97, which implies that the diffusion limitation of propylene by the steric hindrance of naphthalenic species within CHA cavity is more severe than that of ethylene. Indeed, the CHA model implemented in current work only accounts for neutral species, pure silica, and rigid framework, this model would possess some limitations in the construction of force field which is important in considering the diffusion limitation owning to the steric hindrance of guest molecules. A alternative yet more accurate approach is to use the ab intio MD (AIMD) simulations^44,45^, which, however, is very time-consuming. Cnudde et al.^44^ compared the difference between force field MD and AIMD, and found that, despite the values of free energy barriers were quantitatively different, the qualitative trends of these two methods were similar. In this sense, the *D*_empty_/*D*_load_ ratios derived from force field MD, which are 3.34 for ethylene and 3.97 for propylene, can qualitatively reflect that the diffusion limitation of propylene by the steric hindrance of naphthalene species within CHA cavity is more severe than that of ethylene. Based on the above experiments and theoretical calculations, we can find that the naphthalenic species confined within CHA cavity not only serve as the HCPs to preferential formation of ethylene but also further limits the diffusion of large molecules, e.g., propylene. This synergic effect imposed by naphthalenic species can promote the selectivity of ethylene.

**Fig. 5:**
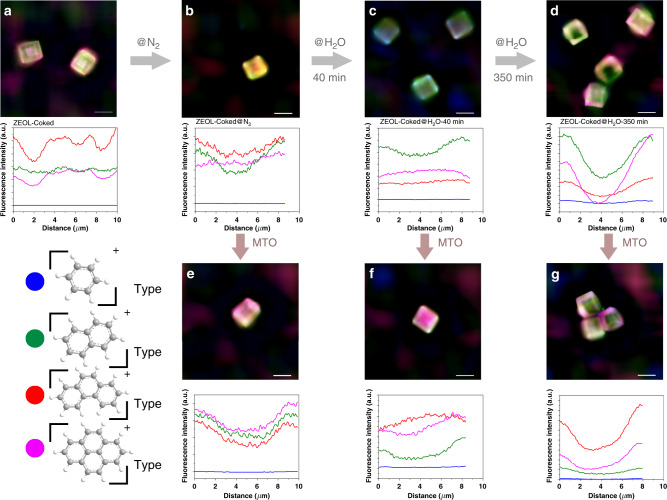
Spatiotemporal evolution of carbonaceous intermediates confined in ZEOL zeolites. **a** ZEOL-Coked sample. **b** ZEOL-Coked@N_2_ sample. **c** ZEOL-Coked@H_2_O-40 min sample **d** ZEOL-Coked@H_2_O-350 min sample. **e**–**g** Deactivated samples of ZEOL-Coked@N_2_, ZEOL-Coked@H_2_O-40 min and ZEOL-Coked@H_2_O-350 min used for MTO reaction, respectively. Scale bar represents 10 μm. The SIM images shown is the fluorescence that originated from the overlap of four profiles with a laser excitation of 405 nm (detection at 435–485 nm, false color: blue), 488 nm (detection at 500–545 nm, false color: green), 561 nm (detection at 570–640 nm, false color: red), 640 nm (detection at 663–738 nm, false color: pink).

Structured illumination microscopy (SIM) was used to resolve the location of carbonaceous species for ZEOL samples. According to the previous experimental work^46–48^ and theorical calculations^49–51^, the excitation wavelengths of B_n_^+^, N_n_^+^, PH_n_^+^, and PYR_n_^+^ are situated around 390, 480, 560, and 640 nm, respectively, and the corresponding emission wavelengths are located in the range of 480–490, 500–520, 620–630, and 670–700 nm, respectively. The wavelengths of illumination and emission detection of SIM used in this work are 405 (detection at 435–485 nm), 488 (detection at 500–545 nm), 561 (detection at 570–640 nm), and 640 nm (detection at 663–738 nm), respectively, which can cover the characteristic area of excitation and emission wavelengths of B_*n*_^+^, N_n_^+^, PH_n_^+^, and PYR_n_^+^. SIM images shown in Fig. 5a clearly elucidate that for ZEOL-Coked sample, the PH_n_^+^ and PYR_n_^+^ mainly locate at the crystal rim and center. And these species located at the crystal rim can block the micropore (Fig. 4d) and hamper the accessibility of activated intermediates (e.g., N_n_^+^) and acid sites inside crystal to the methanol, causing catalyst deactivation. In Fig. 5b, after treatment by nitrogen at 953 K for 40 min, the relative fluorescence intensity of N_n_^+^ increases substantially at especially the crystal rim while the relative intensity of PH_n_^+^ and PYR_n_^+^ decreases. When this sample is evaluated at 723 K for MTO reaction as shown in Fig. 5e, N_n_^+^ deposited at the crystal rim is further transformed into the PH_n_^+^ and PYR_n_^+^. Interestingly, in Fig. 5c, the relatively homogeneous distribution of carbonaceous species throughout the ZEOL-Coked@H_2_O-40 min crystal is revealed. Here the increased fluorescence intensity of N_n_^+^ corresponds to the promotion in naphthalene concentration, which is also shown in Fig. 4a. Unlike the distribution of carbonaceous species over ZEOL-Coked sample performed by original MTO reaction (Fig. 5a), the uniform spatial distribution of carbonaceous species over the ZEOL-Coked@H_2_O-40 min sample after MTO reaction can be observed in Fig. 5f. After a long time (350 min) of steam cracking, the residual amorphous carbon (Supplementary Fig. 16) is mainly concentrated at the crystal rim (Fig. 5d). As shown in Fig. 5g, due to the hindrance of amorphous carbon, the formation of coke species interior SAPO-34 crystal is limited. This diffusion limitation imposed by the amorphous carbon can also be beneficial to improve the initial selectivity of ethylene, while the catalyst lifetime slightly reduces compared with the fresh ZEOL crystal (Supplementary Fig. 11e).

### Coke transformation strategy implemented in a pilot plant

**Fig. 6:**
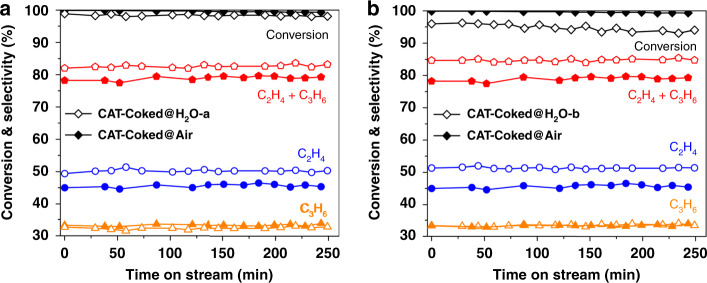
Implementing the coke transformation strategy over CAT catalysts in a pilot plant. **a** MTO reaction performances over the CAT-Coked sample completely regenerated by air combustion at 953 K (abbreviated as CAT-Coked@Air) and treated by steam at 953 K with the water feed of 500 g h^−1^ (abbreviated as CAT-Coked@H_2_O-a). **b** MTO reaction performances over the CAT-Coked@Air sample and CAT-Coked sample treated by steam at 953 K with the water feed of 300 g h^−1^ (abbreviated as CAT-Coked@H_2_O-b).

To attain a reproducible behavior, the coke transformation strategy is evaluated in a fluidized bed reactor-regenerator pilot plant by use of CAT catalyst. The diameters of reactor and regenerator are both 0.124 m, and the corresponding temperatures of MTO reaction and steam cracking are 743 and 953 K for reactor and regenerator, respectively. The schematic diagram and photo of pilot plant are shown in Supplementary Figs. 18, 19. The experimental catalyst circulation rate is 1.2 kg h^−1^. For comparison, we also used air combustion as the regeneration manner to recover the catalyst activity under the same reaction and regeneration temperatures. The light olefins selectivity was 79% in the pilot facility under complete regeneration by air. The corresponding *E*/*P* ratio was 1.36 (Fig. 6). Implementing the coke transformation strategy with steam cracking to the same reaction-regeneration configuration, the light olefins selectivity can reach 83% at methanol conversion of 98% (Fig. 6a) and 85% at methanol conversion of 95% (Fig. 6b). Concomitantly, the *E/P* ratio increases to 1.44 and 1.54, respectively, while the selectivity of propylene remains almost unchanged. The incompletely converted methanol can be separated from gas product and recycled to MTO reactor. Supplementary Fig. 20 shows the composition of flue gas from regenerator, in which only 5 vol% CO_2_ is achieved and H_2_ and CO accounts to 88 vol%. The results under the industrial-alike continuous operation suggest that the proposed coke transformation strategy can significantly promote the formation of light olefins and reduce CO_2_ emissions, bursting the economics and sustainability of MTO process. Considering methanol can readily reach the complete conversion in MTO reaction, and it might be possible that the different ethylene selectivity is due to the excess of zeolite which may reconvert part of the olefins into side products. To this end, we have conducted several experiments in our pilot plant to compare the performance of the air-treated and steam-treated samples at the (almost) same methanol conversion (~ 95%) and similar coke content on catalyst at the reactor inlet (air-treated sample: 7.36 wt%, steam-treated sample: 7.15 wt%). The results are shown in Supplementary Fig. 21. Expectedly, an ethylene selectivity of ~51% has been achieved over steam-treated sample, which is higher than that over air-treated sample (~48%). At such a lower methanol conversion, the influence of excess zeolite can be excluded. However, though the methanol conversion and coke content no doubt are two most important parameters influencing MTO reaction, the back mixing of catalysts and gas will also affect ethylene selectivity to a minor extent because our experiments have been carried out in a fluidized bed reactor-regenerator pilot plant with catalyst circulation. Anyway, from the results of Supplementary Fig. 21, the effects of conversion and coke amount on the ethylene selectivity can be decoupled, and it testified again that steam treatment strategy proposed in this work is more conducive to ethylene formation than conventional air treatment strategy.

## Discussion

We propose a feasible strategy to enhance the ethylene selectivity and carbon atom utilization during MTO reaction by selectively transforming coke species to active carbonaceous intermediates (naphthalenic cations) via steam cracking, which has been verified in both the laboratory-scale reactor and fluidized bed reactor-regenerator pilot plant. With the aid of DFT calculations, together with multiple spectroscopy techniques, e.g., GC-MS, MALDI FT-ICR MS, operando UV-Raman spectra and SIM, the steric stability and catalytic functionality of naphthalenic cations, profound insights of transformation of coke are depicted. Naphthalenic species, confined within CHA cavity not only serve as the active HCPs but also further limit the diffusion of large molecules. This synergic effect imposed by naphthalenic species promotes the selectivity of ethylene. This strategy is much more competitive than current commercial MTO process, with regards to the unexpectedly high light olefins selectivity and low CO_2_ emission. Especially the latter can avoid the emission of greenhouse gas and prevent significant loss of carbon in terms of atom economics. Given the fact that many heterogeneous catalytic reactions are autocatalytic, we expect that this established strategy will also be of high instructive for other heterogeneous catalytic processes.

## Methods

### Computational details

All density functional theory (DFT) calculations were performed with the Gaussian 09 package^52^. Three 36T cluster models representing the structure of CHA zeolite are used to calculate the adsorption energies. Specifically, these three 36T cluster models are respectively (Al_18_P_17_SiO_54_H_36_) representing the negative SAPO-34 zeolite model, (Si_36_O_54_H_36_) representing neutral pure silica model of CHA cavity, and (Al_18_P_17_SiO_54_H_37_) representing SAPO-34 zeolite model containing one Brønsted acid site. These structures were extracted from the periodic structures of CHA. For the extended zeolite model, the terminal Al–H or Si–H was fixed at a bond length of 1.54 Å and oriented along the direction of the corresponding Al–O or Si–O bond. Except H atom of Brønsted acid site, all other H atoms were fixed. In all these three cluster models, Al, P, Si, and O atoms were fully optimized without restrictions. Following the work^8,11,45^, the calculations of adsorption energies based on DFT using the *ω*B97XD and 6-31G (d, p) basis set were conducted. For the calcualtion of adsorption energies, the benzenic (B_n_^+^), naphthalenic (N_n_^+^), phenanthrenic (PH_n_^+^), and pyrenic (PYR_n_^+^) carbocations with *n* methyl substituent were first loaded within negative SAPO-34 zeolite model and neutral pure silica model of CHA cavity, and the corresponding calculated adsorption energies are listed in Supplementary Tables 6 and 7, respectively. The neutral benzene (B_n_), naphthalene (N_n_), phenanthrene (PH_n_), and pyrene (PYR_n_) species with *n* methyl substituent were then loaded within neutral SAPO-34 model containing one Bronsted acid site for calculating the adsorption energies as listed in Supplementary Table 8. The verification of calculated approach is introduced in the Supplementary Information. In this work, we focus on the effect of framework topology rather than framework composition or flexibility^45,53^ on the preferential stabilization of carbenium ions within CHA, and therefore the cluster models were employed in our current work.

For the calculation of transition state, an extended 74 T (SiP_36_Al_37_O_119_H_59_) cluster model extracted from the crystallographic CHA structure represents the structure of neutral SAPO-34 model containing one Brønsted acid site as shown in Supplementary Fig. 6a^54^. For the extended model, the terminal Al–H and P–H were fixed and oriented along the direction of the corresponding Al–O and P–O bond. The location of acid site was chosen at the 8-membered ring, accessible for adsorbents and surrounded by maximum reaction space. To preserve the integrity of the crystalline structure, the 8-membered ring, active center (SiO)_3_-Si–OH–Al–(SiO)_3_ and the adsorbate were set to high-level layer while the rest of atoms were set to low-level layer. The combined theorical ONIOM method was exploited to the prediction of stability of various adsorption structure and geometries of and transition states (TS). For the structure optimization, *ω*B97XD hybrid density function^55^ with 6-31G (d, p) basis sets and semi-empirical AM1 were employed for optimization of structure of high-level and low-level layer, respectively. To obtain high accurate interaction energy, the single-point energies were calculated at the level of *ω*B97XD/6-31G (d, p) on the basis to of optimized structure. The frequency calculations were performed at the same level as geometry optimizations to check whether the saddle point exhibits the proper number of imaginary frequencies. The attained transition state is a first-order saddle point of potential energy surface, with only a single imaginary frequency. The adsorbed state is verified as being situated in the energy minima points of potential energy surface, with only real frequency. The intrinsic free energy was obtained from the *ω*B97XD/6-31G (d, p) total electronic energies and the thermal correction from the *ω*B97XD/6-31G (d, p): AM1 frequency calculations with the correction of zero-point vibration energies. The noncovalent interaction index approach was adopted to visualize the noncovalent interactions between the adsorbate and zeolite framework^56^.

The isosurface plots of the reduced density gradient (RDG) were obtained by calculating the RDG functions, defined as RDG(*r*) = 1/(2(3π^2^)^1/3^)|∇*ρ*(*r*)|/(*ρ*(*r*)^4/3^) (*ρ* represents the electron density) and the quantity sign(*λ*_2_)*ρ* (sign(*λ*_2_)*ρ* < 0, H-bonding interaction; sign(*λ*_2_)*ρ* ≈ 0, weak van der Waals interaction; sign(*λ*_2_)*ρ* > 0, strong repulsive interaction) by the Multiwfn software^57^.

The molecular diffusivities were obtained from molecular dynamics (MD) simulations. MD simulations were carried out using the Materials Studio simulation package (Accelrys Software). The adsorption of molecules in CHA structure were performed using the grand canonical Monte Carlo (GCMC) simulation method. Periodic boundary conditions were applied in all three directions. The interatomic interactions were described by the condensed-phase-optimized molecular potentials for atomistic simulation studies (COMPASS) force field. The applicability of the used force field was checked in the previous work^51^. The Ewald & Group summation method has an Ewald accuracy of 10^−5^ kcal mol^−1^ when it is used for calculating electrostatic potential energy. To achieve an equilibrium state, 10^7^ Monte Carlo steps were carried out. The zeolitic framework with a rigid structure was considered. The metropolis scheme was used at a constant loading and constant temperature. To minimize the energy of constructed structures, all the structures were equilibrated by five anneal cycles from 250 to 750 K with a heating ramp of five to refine the conformation. Dynamics processes in the NVT ensemble, i.e., the number of particles (N), volume (V), and temperature (T) as the constants, were performed for 10,000 ps in 10,000,000 steps for ethylene and 20,000 steps in 20,000,000 steps for propylene 723 K. The velocity Verlet algorithm was used to integrate the Newton’s equations of motion with a time step of 1 fs. A cutoff radius of 18.5 Å was assumed for Lennard–Jones interaction potential calculation. The simulated temperature was controlled by a Nosé thermostat. The structures considered in this study and the mean square displacement (MSD) of ethylene and propylene are shown in Supplementary Fig. 14. The slope of MSD as a function of time was used to determine the self-diffusivity following Einstein relation:^58^

1 $${\rm{MSD}}\left( \tau \right) = 2nD\tau + b,$$

where *n* is the dimension of framework (*n* = 1, 2, and 3 for 1D, 2D, and 3D frameworks, respectively) and *b* the thermal factor arising from atomic vibrations.

### Catalyst preparation

Commercial shaped SAPO-34 catalyst (CAT), which contains catalytically active SAPO-34 zeolites dispersed in the matrix and binder, and corresponding SAPO-34 zeolites (ZEOS) under study were purchased from Catalyst & Catalysis Technology Co. Ltd. of CAS. The synthesis procedures of the SAPO-34 zeolites with large crystal size (ZEOL) were taken from existing recipes from the open literature^59^.

### Catalyst characterization

The powder XRD patterns were recorded on a PANalytical X’ Pert PRO X-ray diffractometer with Cu-K_*α*_ radiation (*λ* = 1.54059 Å), operating at 40 kV and 30 mA. XRD patterns were recorded in the range of 2*θ* = 5–40°.

The quantitative elemental analysis was determined by XRF spectrometer (Philips, Magix-601).

The morphology and crystal size of SAPO-34 samples used in this work were observed by scanning electron microscopy (Hitachi TM3000). The morphology of insoluble carbonaceous species was obtained by Field emission scanning electron microscopy (FESEM, Hitachi SU8020).

Temperature-programmed desorption of ammonia (NH_3_-TPD) was measured on a Micrometric 2920 chemical adsorption instrument to enumerate the acid site density of the SAPO-34 samples. Firstly, sample was loaded into a quartz U-shaped reactor and pretreated at 873 K for 1 h under helium atmosphere. Then, the sample was cooled to 373 K and saturated with NH_3_. Lastly, NH_3_-TPD was carried out in a constant flow of helium atmosphere (20 mL min^−1^) from 373 to 873 K at a heating rate of 10 K min^−1^.

The nitrogen adsorption–desorption measurements were conducted with Micromeritics ASAP 2020 at 77 K after SAPO-34 samples degassed at 623 K under vacuum for 6 h to obtain their textural properties.

The coke contents of SAPO-34 samples were measured by thermo-gravimetric analysis (TGA) carried out on a SDTQ 600 setup at the temperature range of 323–1173 K with a heating rate of 15 K min^−1^ under an air flow of 100 mL min^−1^. The coke content is defined as the quantity of carbonaceous species in per unit mass of calcined catalyst and the loss of mass between 573 and 1173 K measured by TGA was used to estimate the coke content.

Gas chromatography-mass spectrometry (GC-MS) and matrix-assisted laser desorption ionization Fourier-transform ion cyclotron resonance (MALDI FT-ICR) MS were used to identify the carbonaceous species. Fifteen milligram of the coked SAPO-34 sample was dissolved in 1 mL 20 wt% hydrofluoric acid in a Teflon container. The organic compounds were extracted by the addition of 1 mL dichloromethane containing 100 ppm hexachloroethane as internal standard. The organic components in extracted phase with molecular weight smaller than 200 were analyzed by GC-MS equipped with a HP-5 capillary column and FID detector. Then, the extracted phase was mixed with 1,8,9-anthracenetriol (dithranol) matrix. Lastly, 1 μL of the mixture was taken to spot on the sample holder. The 15-T FT-ICR MS (Bruker Daltonics, Bremen, Germany) equipped with a Nd: YAG laser emitting 355 nm laser to generate ions and a time-of-flight mass analyzer in reflection mode was used to record MS signals. Ion source parameters were optimized to a broadband range (150 < *m*/*z* < 2000), which allowed the analysis of carbonaceous species with molecular weight greater than 200 Da.

Diffuse reflectance infrared Fourier transform spectroscopy (DRIFT) spectra were collected on a Bruker Vextex 27 spectroscope supplied with MCT detector^60^. For each experiment, the same quantity of SAPO-34 sample was preheated in nitrogen atmosphere at 623 K for 1 h in the diffuse reflectance infrared cell with ZnSe window to remove adsorbed water. The absorption spectra were obtained by collection 16 scans at 4 cm^−1^ resolution at 623 K.

### MTO reaction and steam cracking

To remove the organic template, all SAPO-34 samples were firstly calcinated at 873 K for 6 h. In MTO reaction, the mass ratio of water to methanol in the feed was 0.2. ZEOS zeolite was pressed into flakes without binder at 10 MPa, and then crushed into pellets and sieved to a diameter of 250–425 μm. To keep the intact morphology of ZEOL for subsequent use in structured illumination microscopy, the prilling of ZEOL zeolite was not implemented. Then MTO reactions over ZEOS and ZEOL zeolites with loading of 0.100 g were carried out in a quartz tubular fixed bed reactor with the inner diameter of 0.004 m under atmospheric pressure with the weight hour space velocity (WHSV) of 6.6 g_MeOH_ g_cat._^−1^ h^−1^ at 723 K. When the methanol conversion decreased to ~80%, under the sweeping of nitrogen atmosphere, ZEOS and ZEOL samples deposited with coke (ZEOS-Coked and ZEOL-Coked) were heated at a rate of 5 K min^−1^ to 953 K and aged for 40 min (ZEOS-Coked@N_2_ and ZEOL-Coked@N_2_). Then ZEOS-Coked@N_2_ and ZEOL-Coked@N_2_ samples were treated by steam at 953 K (ZEOS-Coked@H_2_O and ZEOL-Coked@H_2_O) for different times with WHSH of 2.6 g_Steam_ g_cat._^−1^ h^−1^. Subsequently, these samples were cooled to 723 K, and MTO reactions were catalyzed with the same WHSV, i.e., 6.6 g_MeOH_ g_cat._^−1^ h^−1^, and composition of reactant feed.

MTO reaction over CAT catalyst was carried out in a micro fluidized bed with an inner diameter of 0.019 m and a height of 0.35 m under atmospheric pressure with the WHSV of 2 g_MeOH_ g_cat_.^−1^ h^−1^ at 723 or 763 K, and 5.0 g of CAT catalyst was loaded into the reactor. When MTO reaction proceeded for a period of time and the methanol conversion was less than 100%. Similarly, under the sweeping of nitrogen atmosphere, the CAT catalyst deposited with coke (CAT-Coked) was heated at a rate of 5 K min^−1^ to 953 K and aged for 40 min (CAT-Coked@N_2_). Then the CAT-Coked@N_2_ sample was treated by steam at 953 K (CAT-Coked@H_2_O) for different times, i.e., 5, 10, 20, and 180 min, with WHSV of 3.0 g_Steam_ g_cat._^−1^ h^−1^. Subsequently, these samples were cooled to 723 or 763 K, and MTO reactions were catalyzed with the same WHSV and composition of reactant feed.

The diameters of fluidized bed reactor and regenerator in the circulating configuration were both 0.124 m, experimental catalyst circulation rate is 1.2 kg h^−1^. The temperatures of MTO reaction and steam cracking were 743 and 953 K, respectively. Note, the liquid flow of methanol (99.5% purity) or deionized water was passed through a vaporizer before entering the reactor to guarantee well vaporization of liquid feed.

The effluent products of MTO reaction and steam cracking were analyzed by an online Agilent 7890 A GC equipped with two detectors. The organic compositions were monitored using a Plat-Q capillary column connected to a flame ionization detector (FID) and the CO, CO_2_, and H_2_ were determined suing a TDX-01 packed column connected to a thermal conductivity detector (TCD). The methanol conversion and olefins selectivity were calculated on CH_2_ basis. Dimethyl ether (DME) was considered as reactant in the calculation.

### Operando UV-Raman spectra

Operando UV-Raman spectra was collected using a home-build spectrometer^38^. The system was composed of a 257 nm constant-wave laser, a 25 mm diameter off-axis parabolic mirror as the light-collecting element, an edge filter to filter Rayleigh scattered light, a spectrograph, and a UV-CCD camera produced by Andor. All spectra were calibrated by placing the main Raman peak of monocrystalline Si at 520 cm^−1^. To ensure optical throughput, the slit width was set at 150 μm, resulting in a spectral resolution of ∼7 cm^−1^. For most experiments, the laser power at the sample was kept below 2 mW to prevent burning effects. The typical accumulation time per spectrum was ∼30 s. For operando UV-Raman experiments, 10 mg of the ZEOS-Coked sample were treated by steam at 923 K for 2 h, the WHSV was of 2.0 g_Steam_ g_cat._^−1^ h^−1^.

### Structured illumination microscopy (SIM)

SIM was applied to gain super-resolution images on the spatiotemporal distribution of the coke species located in ZEOL series samples. The super-resolution imaging was carried out using a Nikon N-SIM super-resolution microscopy system with a motorized inverted microscopy ECLIPSE Ti2-E, a 100×/NA 1.49 oil immersion TIRF objective lens (CFI HP) and ORCA-Flash 4.0 sCMOS camera (Hamamatsu Photonics K.K.)^61,62^. Imaging with 405, 488, 561, and 640 nm multilaser light sources, the emissions by excitation were collected with four photomultiplier tubes in the range 435–485, 500–545, 570–640, 663–738 nm for the four lasers, respectively. Imaging was performed under ex-situ condition at room temperature. ZEOL series samples were loaded on glass-bottomed culture dishes (35 mm dish with 20 mm well) and images were taken at a *Z*-plane of middle of zeolitic crystal. The software NIS-Elements Ar and N-SIM Analysis were used to analyze the collected images and computationally reconstruct the super-resolution image.

## Supplementary information

Supplementary Information

## Data Availability

The data that support the findings of this study are available from the corresponding author M.Y. upon reasonable request.

## Code availability

The code used in this paper is available from the corresponding authors M.Y. upon reasonable request.
