# Supplementary material for: Directed transforming of coke to active intermediates in methanol-to-olefins catalyst to boost light olefins selectivity
Source: Nat Commun. 2021 Jan 4;12:17. doi: 10.1038/s41467-020-20193-1 (PMC7782712; doi:10.1038/s41467-020-20193-1)
Supplement: Supplementary file 1 — Supplementary Information [file 41467_2020_20193_MOESM1_ESM.pdf]

Supplementary Information

Directed transforming of coke to active intermediates in methanol-to-olefins catalyst to boost light olefins selectivity

Zhou et.al

Supplementary Table 1. Sample symbols and their preparation methods used in this work.

**Samples**	**Preparation method / sources**
ZEOL	Fresh SAPO-34 zeolite with large crystal size
ZEOS	Fresh SAPO-34 zeolite with small crystal size
CAT	Catalyst pellet composed of ZEOS zeolites, matrix and binder
ZEOS@H_2O-180min	ZEOS sample treated by steam at 953 K for 180 minutes
ZEOS-Coked	Deactivated ZEOS sample via MTO reaction at 723 K for 175 minutes
ZEOS-Coked@H_2O-180min@Air	ZEOS-Coked sample treated by steam at 953 K for 180 minutes and then calcinated by air at 923 K for 300 minutes
ZEOS-Coked@N_2	ZEOS-Coked sample treated by nitrogen at 953 K for 40 minutes
ZEOS-Coked@H_2O-xxmin	ZEOS-Coked@N_2 samples treated by steam at 953 K for different times
ZEOL-Coked	Deactivated ZEOL sample via MTO reaction at 723 K for 35 minutes
ZEOL-Coked@N_2	ZEOL-Coked sample treated by nitrogen at 953 K for 40 minutes
ZEOL-Coked@H_2O-xxmin	ZEOL-Coked@N_2 samples treated by steam at 953 K for different times
CAT-Coked	Deactivated CAT sample via MTO reaction at 723 K for 180 minutes or 763 K for 110 minutes
CAT-Coked@N_2	CAT-Coked sample treated by nitrogen at 953 K for 40 minutes
CAT-Coked@H_2O-xxmin	CAT-Coked@N_2 samples treated by steam at 953 K for different times

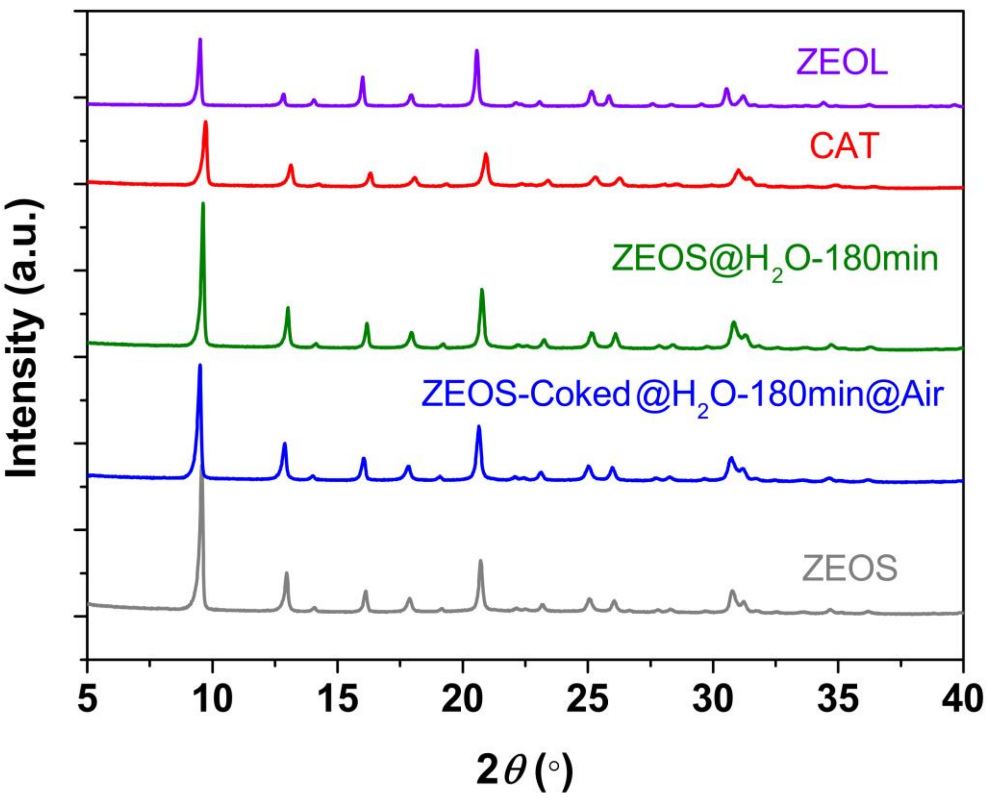

Supplementary Figure 1. Powder XRD patterns of ZEOL, CAT, ZEOS, ZEOS@H_2O-180min and ZEOS-Coked@H_2O-180min@Air.

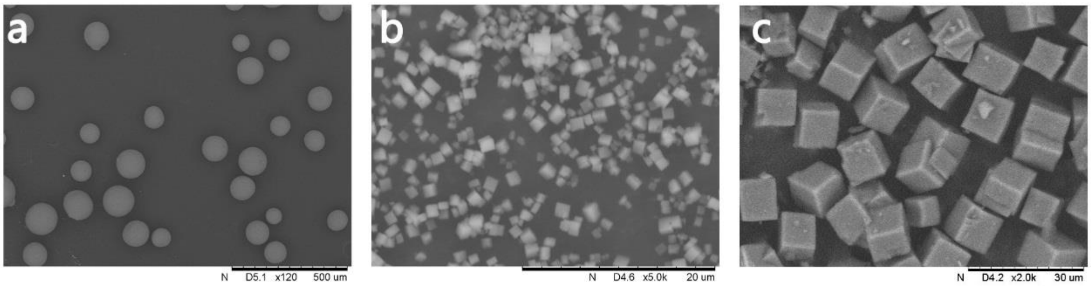

Supplementary Figure 2. SEM images of (**a**) CAT, (**b**) ZEOS and (**c**) ZEOL samples. CAT sample exhibits a spherical particle morphology and has an average particle size of ~ 90 μm. ZEOS and ZEOL samples both exhibit cubic morphologies, while ZEOS sample has an average crystal size of ~ 1 μm and ZEOL of ~ 10 μm.

Supplementary Table 2. Bulk elemental composition of ZEOS and ZEOL samples determined from XRF.

Sample	Elemental composition
ZEOS	$(Si_{0.07}Al_{0.464}P_{0.466})O_2$
ZEOL	$(Si_{0.08}Al_{0.487}P_{0.433})O_2$

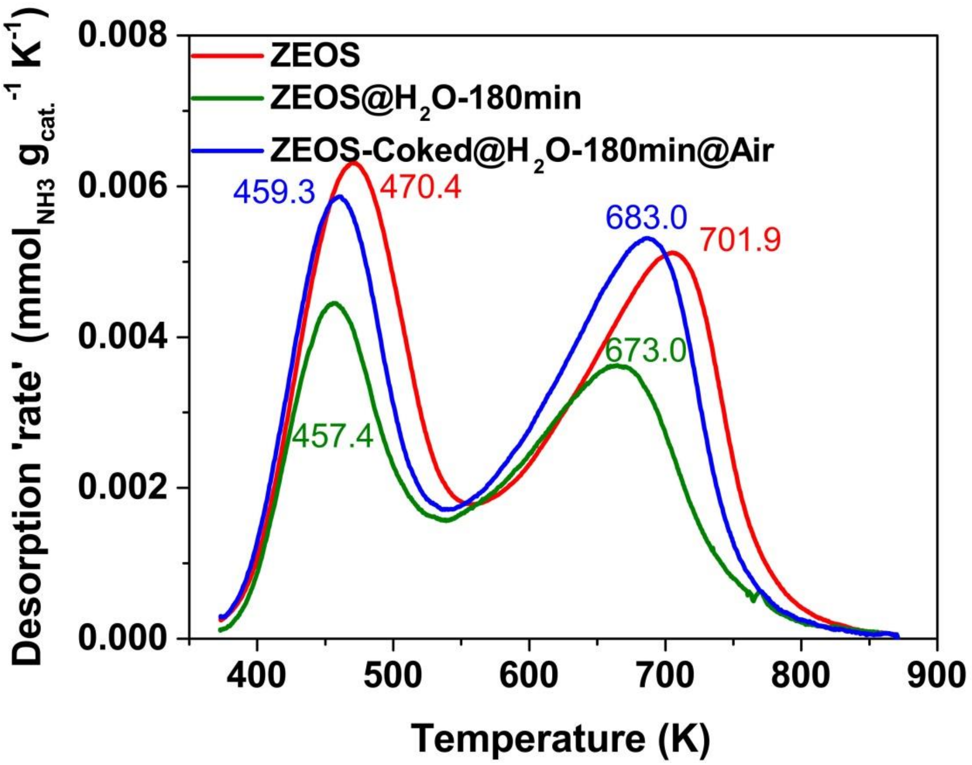

Supplementary Figure 3. NH_3-TPD profiles of ZEOS, ZEOS@H_2O-180min and ZEOS-Coked@H_2O-180min@Air samples. The TPD profiles are plotted as the desorption 'rate' versus temperature. A low-temperature desorption peak at ca. 373-573 K and a high-temperature peak ca. 573-873 K are assigned to the desorption of NH_3 from weak/medium and strong acid sites, respectively. In particular, the strong acid sites are generally resulted from the bridge hydroxyl groups Si(OH)Al, i.e. Brønsted acid site, of SAPO molecular sieves.

Supplementary Table 3. Acid site density of SAPO-34 samples determined from the area calculated under the NH_3-TPD profiles. Peak-differentation-imitating analysis by Gaussian function was used to deconvolution the NH_3-TPD profiles shown in Supplementary Figure 3.

Sample	Brønsted (mmol $g_{cat.}^{-1}$)	Weak/medium (mmol $g_{cat.}^{-1}$)	Total (mmol $g_{cat.}^{-1}$)
ZEOS	0.75	0.65	1.41
ZEOS@H_2O-180min	0.47	0.58	1.05
ZEOS-Coked@H_2O-180min@Air	0.75	0.52	1.27

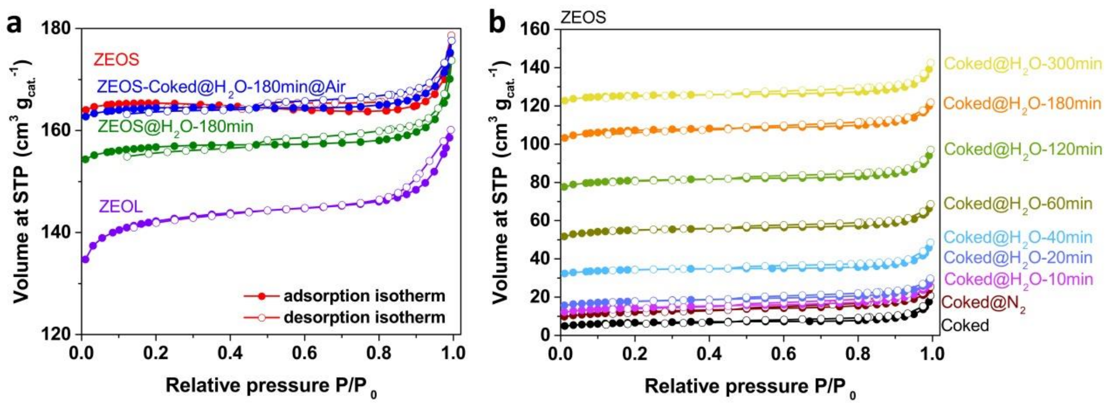

Supplementary Figure 4. Nitrogen adsorption and desorption isotherms at 77 K of (**a**) ZEOS, ZEOS@H_2O-180min, ZEOS-Coked@H_2O-180min@Air and ZEOL; (**b**) ZEOS-Coked, ZEOS-Coked@N_2, ZEOS-Coked@H_2O-10, 20, 40, 60, 120, 180 and 300min.

Supplementary Table 4. Textural properties obtained from nitrogen adsorption and desorption isotherms presented in Supplementary Figure 4.

Sample	Surface area (m^2 $g_{cat.}^{-1}$)		Pore volume (cm^3 g_{cat}^{-1})	
	S_{total}[a]	S_{micro}[b]	V_{total}[c]	V_{micro}[b]
ZEOS	520	518	0.26	0.26
ZEOS-Coked	22	10	0.02	0.01
ZEOS-Coked@N_2	41	22	0.03	0.01
ZEOS-Coked@H_2O-10min	47	30	0.04	0.02
ZEOS-Coked@H_2O-20min	58	42	0.04	0.02
ZEOS-Coked@H_2O-40min	109	95	0.06	0.05
ZEOS-Coked@H_2O-60min	176	157	0.10	0.08
ZEOS-Coked@H_2O-120min	257	238	0.14	0.12
ZEOS-Coked@H_2O-180min	340	316	0.18	0.16
ZEOS-Coked@H_2O-300min	396	381	0.21	0.19
ZEOS@H_2O-180min	495	481	0.25	0.24
ZEOS-Coked@H_2O-180min@Air	518	510	0.26	0.25
ZEOL	455	417	0.24	0.20

[a]Total surface area is determined by BET equation; [b]Micropore surface area, external surface area and micropore volume are calculated by t-plot method; [c]Mesopore volume is determined by BJH method.

Supplementary Table 5. Coke content of ZEOS-Coked samples treated by nitrogen for 40 min and by steam for different times at 953 K.

Sample	Coke content (wt%)
ZEOS-124min	16.46
ZEOS-Coked	19.47
ZEOS-Coked@N_2	18.56
ZEOS-Coked@H_2O-10min	18.07
ZEOS-Coked@H_2O-20min	17.07
ZEOS-Coked@H_2O-40min	14.99
ZEOS-Coked@H_2O-60min	12.66
ZEOS-Coked@H_2O-120min	10.44
ZEOS-Coked@H_2O-180min	8.26
ZEOS-Coked@H_2O-300min	5.11

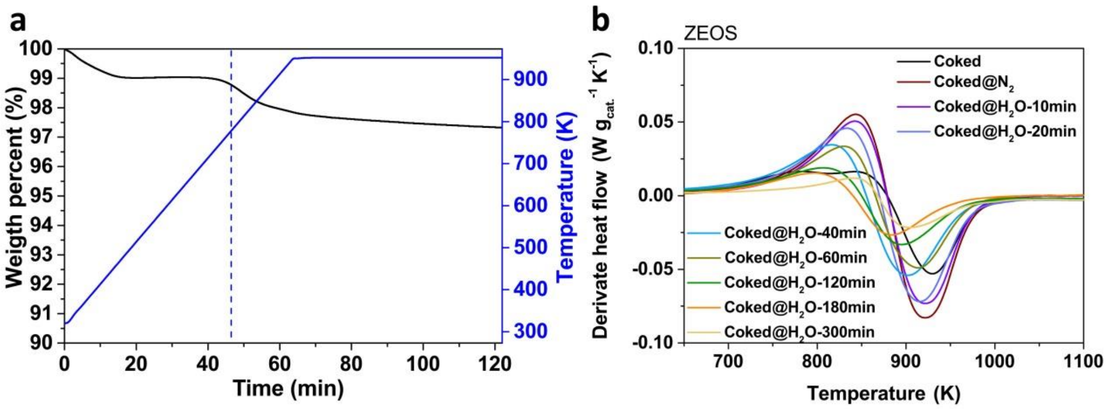

Supplementary Figure 5. (**a**) TGA curve of ZEOS-Coked sample swept by nitrogen from 323 K to 953 K. (**b**) Differential thermogravimetry profiles of ZEOS-Coked samples treated by nitrogen for 40 min and by steam for different times at 953 K.

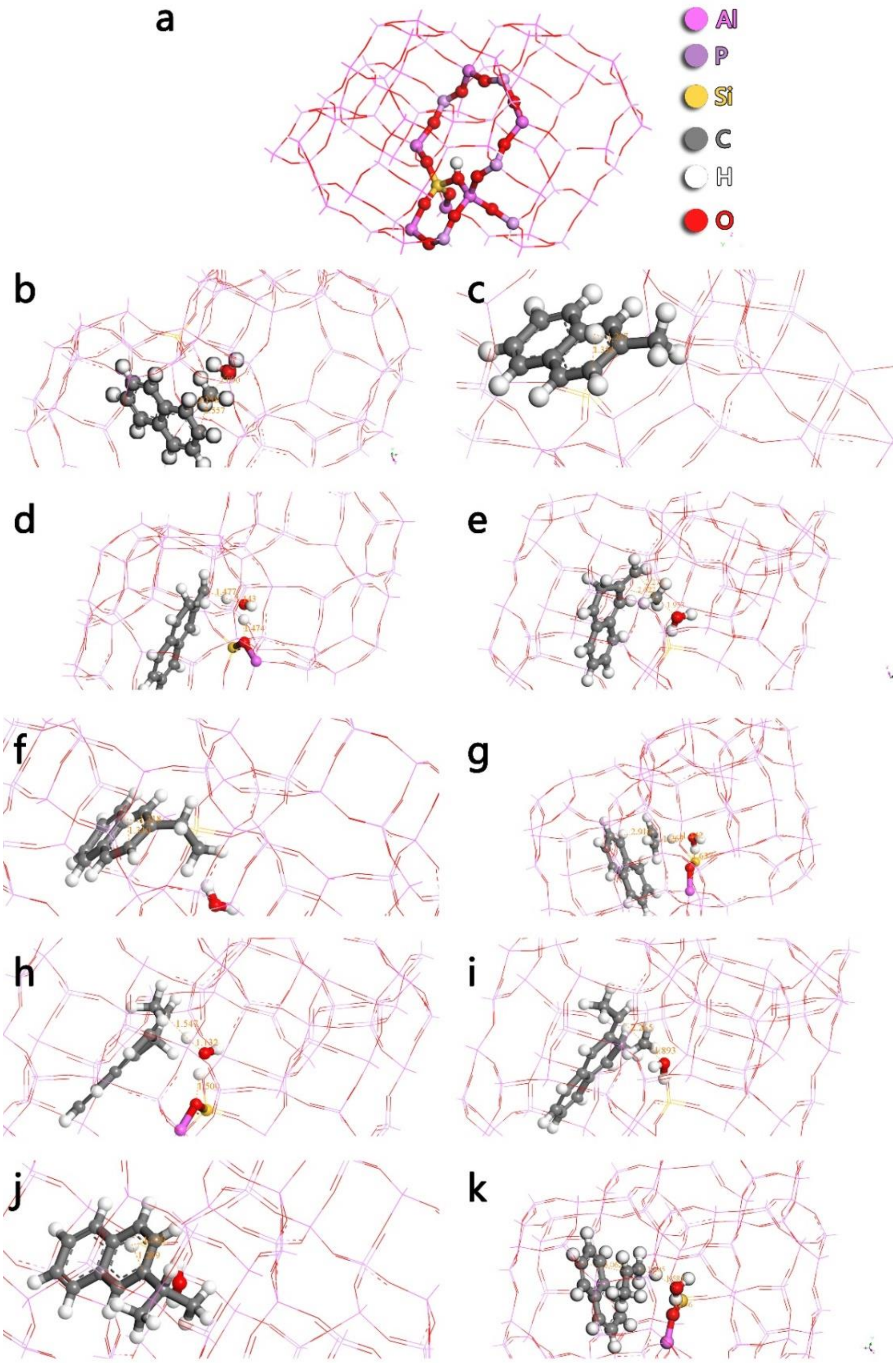

Supplementary Figure 6. (**a**) The used 74T cluster, representing SAPO-34, with 26T high level and one acid site indicated. Transition state of (**b**) M1, (**c**) S_H1, (**d**) DP1, (**e**) M2, (**f**) SH2, (**g**) E1, (**h**) DP2, (**i**) M3, (**j**) S_H3 and (**k**) E2 reaction step based on the proposed side-chain mechanism in SAPO-34 starting from Naphthalene. Distance are indicated in Å.

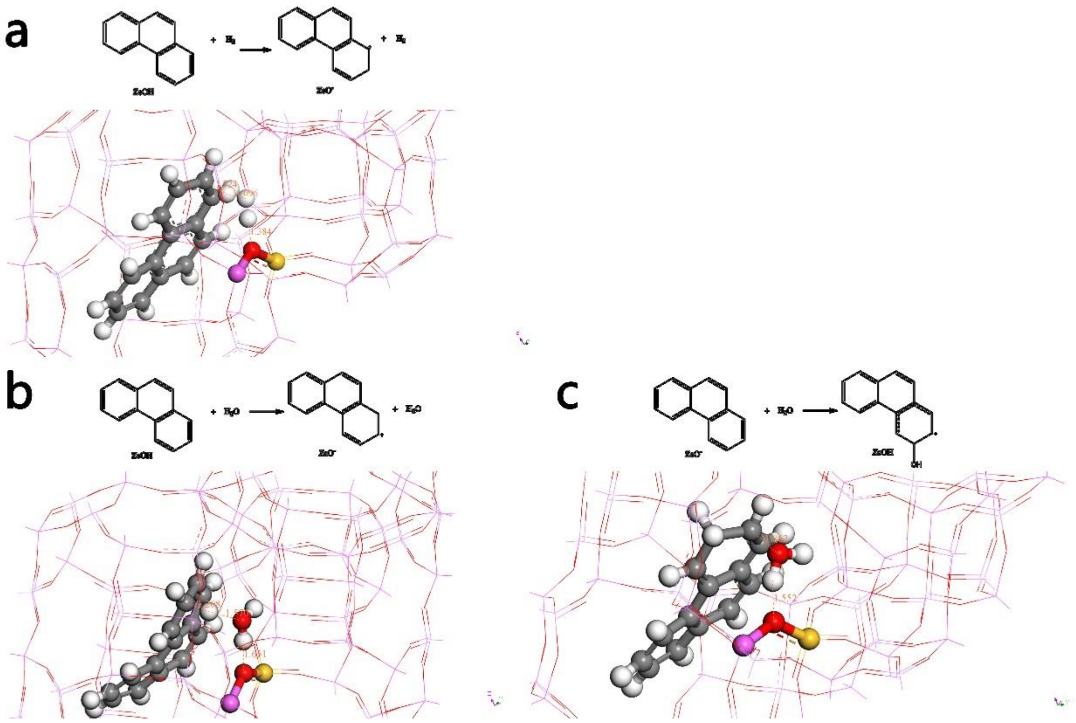

Supplementary Figure 7. Transition state of protonation of phenanthrene by (**a**) hydrogen and (**b**) water, and the hydroxylation of phenanthrene by (**c**) water in SAPO-34. Distance are indicated in Å.

To validate the reliability of the DFT calculations, the adsorption free energies (ΔG_{ads}) of gempentamethyl-benzenic (gem-B_5^+) and gemheptamethyl-benzenic (gem-B_7^+) carbocations confined within neutral pure silica model of CHA cavity were calculated to be -168.93 and -62.03 kJ mol^{-1} at 673 K, which shows the stability of gem-B_5^+ is higher than that of gem-B_7^+ in CHA cavity. This is consistent with the calculations by Ferri et al[1]. Owing to the framework flexibility of SAPO zeolites, carbenium ions confined within SAPO zeolites cavity will lead to the expansion of cavity[2]. The composition of framework can affect the preferential stabilization of carbenium ions. To verify the reliability of DFT calculations with the used cluster models in this work, the effect of framework composition on interaction energies E_{int} were also compared with results of periodic DFT calculations by Ferri et al.[3]. In this work, the interaction energies of gem-B_5^+ and gem-B_7^+ within SAPO-34 zeolite model are -114.42 and -92.19 kJ mol^{-1} ($E_{int(7/5)}$ = 0.81), and the E_{int} of gem-B_5^+ and gem-B_7^+ within neutral pure silica model of CHA cavity are -74.41 and -54.01 kJ mol^{-1} ($E_{int(7/5)}$ = 0.73). These calculated results are consistent with the results of periodic DFT calculations[3].

Supplementary Table 6. The adsorption enthalpies, adsorption entropies and adsorption free energies at 953 K of carbenium ions within negative SAPO-34 zeolite model.

Carbocations (X^+-ZEO^-)	ΔH_{ads} (kJ mol $^{-1}$)	ΔS_{ads} (J mol^{-1} K^{-1})	ΔG_{ads} (953 K) (kJ mol $^{-1}$)
B_0^+	-385.05	-150.76	-241.36
B_1^+	-377.00	-164.58	-220.14
B_2^+	-387.16	-163.46	-231.36
B_3^+	-369.73	-202.19	-177.01
B_4^+	-383.86	-198.45	-194.71
B_5^+	-332.54	-214.48	-128.11
B_6^+	-370.71	-226.25	-155.07
gem-B_5^+	-467.60	-194.94	-281.79
gem-B_7^+	-369.21	-231.41	-148.64
N_0^+	-406.29	-172.68	-241.70
N_1^+	-398.29	-191.00	-216.24
N_2^+	-414.54	-190.84	-232.68
PH_0^+	-376.30	-196.62	-188.89
PYR_0^+	-357.40	-184.70	-181.36

B_n^+, N_n^+, PH_n^+ and PYR_n^+ stand for benzenic, naphthalenic, phenanthrenic and pyrenic carbocation with *n* methyl substituents, respectively.

Supplementary Table 7. The adsorption enthalpies, adsorption entropies and adsorption free energies at 953 K of carbenium ions within neutral pure silica model of CHA cavity.

Carbocations (X^+-ZEO)	ΔH_{ads} (kJ mol^{-1})	ΔS_{ads} (J mol^{-1} K^{-1})	ΔG_{ads} (953 K) (kJ mol^{-1})
B_0^+	-204.11	-158.45	-53.09
B_1^+	-200.08	-150.32	-56.80
B_2^+	-209.15	-156.04	-60.42
B_3^+	-214.25	-166.09	-55.94
B_4^+	-220.98	-175.17	-54.02
B_5^+	-251.45	-207.38	-53.79
B_6^+	-239.88	-208.32	-41.32
gem-B_5^+	-299.00	-193.22	-114.83
gem-B_7^+	-211.27	-221.70	0.05
N_0^+	-224.43	-163.42	-68.67
N_1^+	-223.40	-175.77	-55.87
N_2^+	-223.08	-189.17	-52.77
PH_0^+	-218.14	-207.23	-20.62
PYR_0^+	-220.16	-212.97	-17.17

B_n^+, N_n^+, PH_n^+ and PYR_n^+ stand for benzenic, naphthalenic, phenanthrenic and pyrenic carbocation with *n* methyl substituents, respectively.

Supplementary Table 8. The adsorption enthalpies, adsorption entropies and adsorption free energies at 953 K of neutral carbonaceous species within neutral SAPO-34 model containing Bronsted acid site.

Carbonaceous species (X-ZEO)	ΔH_{ads} (kJ mol $^{-1}$)	ΔS_{ads} (J mol^{-1} K^{-1})	ΔG_{ads} (953 K) (kJ mol $^{-1}$)
B_0	-62.01	-115.29	47.88
B_1	-86.92	-173.45	78.41
B_2	-94.31	-170.33	68.05
B_3	-95.83	-185.80	81.27
B_4	-100.67	-199.68	89.66
B_5	-84.80	-192.47	98.65
B_6	-99.03	-190.08	82.14
N_0	-111.55	-159.38	40.37
N_1	-113.79	-183.61	61.21
N_2	-130.21	-184.81	45.94
PH_0	-116.36	-198.96	73.28
PYR_0	-83.05	-188.03	96.17

B_n, N_n, PH_n and PYR_n stand for benzene, naphthalene, phenanthrene and pyrene species with *n* methyl substituents, respectively.

The intrinsic free energy barrier and reaction rate constant were determined by the changes in free energies ($\Delta G^{\ddagger}$), enthalpies ($\Delta H^{\ddagger}$) and entropies ($\Delta S^{\ddagger}$), which are obtained from the ωB97XD/6-31G (d, p) total electronic energies and the thermal correction from the ωB97XD/6-31G (d, p): AM1 frequency calculations with the correction of zero-point vibration energies. The free energy barriers are defined as the energy difference between the reactant and transition state (TS), and the reaction free energies are the energy difference between reactant and product, all of them for the same reaction step. The reaction rate constant k (s^{-1}) was calculated by using classical transition-state theory (TST) by the equation

$$k = \frac{k_B T}{h} exp\left(\frac{\Delta S^{\ddagger}}{R}\right) exp\left(-\frac{\Delta H^{\ddagger}}{RT}\right). \quad (1)$$

where k_B is the Boltzmann constant (J K^{-1}), h is the Planck constant (J s), R is the ideal gas constant (J mol^{-1} K^{-1}), T is the absolute temperature (K), $\Delta H^{\ddagger}$ and $\Delta S^{\ddagger}$ are the changes of standard molar enthalpy (J mol^{-1}), and entropy (J mol^{-1} K^{-1}) between the reactants and the transition state, respectively.

Supplementary Table 9. Calculated kinetic results of free energy barriers ($\Delta G^{\ddagger}$), relative reaction rate constant (k), enthalpy barriers ($\Delta H^{\ddagger}$) and entropy losses ($-T\Delta S^{\ddagger}$), and thermodynamic results of reaction free energies (ΔG_r), reaction enthalpies (ΔH_r) and reaction entropies ($-T\Delta S_r$) at 723 K of each step for the formation of ethylene and propylene based on the proposed side-chain mechanism in SAPO-34 starting from naphthalene.

step	kinetics				thermodynamics		
	$\Delta G^{\ddagger}$ (kJ mol^{-1})	k (s^{-1})	$\Delta H^{\ddagger}$ (kJ mol^{-1})	$-T\Delta S^{\ddagger}$ (kJ mol^{-1})	ΔG_r (kJ mol^{-1})	ΔH_r (kJ mol^{-1})	$-T\Delta S_r$ (kJ mol^{-1})
				Ethylene formation			
M1	145.60	4.56×10^{2}	124.75	20.85	94.25	79.78	14.47
S_H1	49.21	4.20×10^{9}	49.96	-0.75	-9.24	-12.45	3.21
DP1	85.05	1.08×10^{7}	69.61	15.43	87.74	85.12	2.62
M2	164.44	1.98×10^{1}	126.80	37.64	-69.02	-81.72	12.70
S_H2	52.45	2.45×10^{9}	50.59	1.86	-56.78	-39.49	-17.28
E1	55.06	1.58×10^{9}	51.01	4.06	-65.12	-35.60	-29.52
				Propylene formation			
DP2	98.18	1.22×10^{6}	80.91	17.27	82.26	96.09	-13.83
M3	101.42	7.08×10^{5}	79.46	21.96	-94.73	-118.25	23.52
S_H3	37.15	3.12×10^{10}	44.98	-7.82	-43.76	-17.59	-26.17
E2	29.86	1.05×10^{11}	38.09	-8.23	-68.28	-35.21	-33.06

M1 represents methylation, S_H represents hydrogen shift, DP1 represents deprotonation and E represents elimination.

Supplementary Table 10. Calculated reaction rate constant (*k*) at 623, 673, 723 and 773 K of each step for the formation of ethylene and propylene based on the proposed side-chain mechanism in SAPO-34 starting from naphthalene.

Step	*k* (623 K) (s^{-1})	*k* (673 K) (s^{-1})	*k* (723 K) (s^{-1})	*k* (773 K) (s^{-1})
		Ethylene formation		
M1	0.16×10^{2}	0.97×10^{2}	4.56×10^{2}	17.44×10^{2}
S_H1	1.11×10^{9}	2.26×10^{9}	4.20×10^{9}	7.18×10^{9}
DP1	0.17×10^{7}	0.46×10^{7}	1.08×10^{7}	2.28×10^{7}
M2	0.07×10^{1}	0.41×10^{1}	1.98×10^{1}	7.76×10^{1}
S_H2	0.63×10^{9}	1.31×10^{9}	2.45×10^{9}	4.22×10^{9}
E1	0.41×10^{9}	0.84×10^{9}	1.58×10^{9}	2.74×10^{9}
		Propylene formation		
DP2	0.14×10^{6}	0.45×10^{6}	1.22×10^{6}	2.90×10^{6}
M3	0.85×10^{5}	2.65×10^{5}	7.08×10^{5}	16.66×10^{5}
S_H3	0.94×10^{10}	1.79×10^{10}	3.12×10^{10}	5.56×10^{10}
E2	0.38×10^{11}	0.65×10^{11}	1.05×10^{11}	1.58×10^{11}

Supplementary Table 11. Calculated kinetic results of free energy barriers ($\Delta G^{\ddagger}$), relative reaction rate constant (k), enthalpy barriers ($\Delta H^{\ddagger}$) and entropy losses ($-T\Delta S^{\ddagger}$), and thermodynamic results of reaction free energies (ΔG_r), reaction enthalpies (ΔH_r) and reaction entropies ($-T\Delta S_r$) at 953 K of element step for the protonation and hydroxylation of phenanthrene by hydrogen and water.

step	kinetics				thermodynamics		
	$\Delta G^{\ddagger}$ (kJ mol^{-1})	k (s^{-1})	$\Delta H^{\ddagger}$ (kJ mol^{-1})	$-T\Delta S^{\ddagger}$ (kJ mol^{-1})	ΔG_r (kJ mol^{-1})	ΔH_r (kJ mol^{-1})	$-T\Delta S_r$ (kJ mol^{-1})
P(H_2)	220.47	1.24×10^{1}	155.40	-65.08	94.96	91.09	-3.87
P(H_2O)	110.11	1.39×10^{7}	99.64	-10.48	101.48	122.47	21.00
HY(H_2O)	102.43	3.66×10^{7}	53.719	-48.71	86.65	38.73	-47.91

P represents protonation and HY represents hydroxylation.

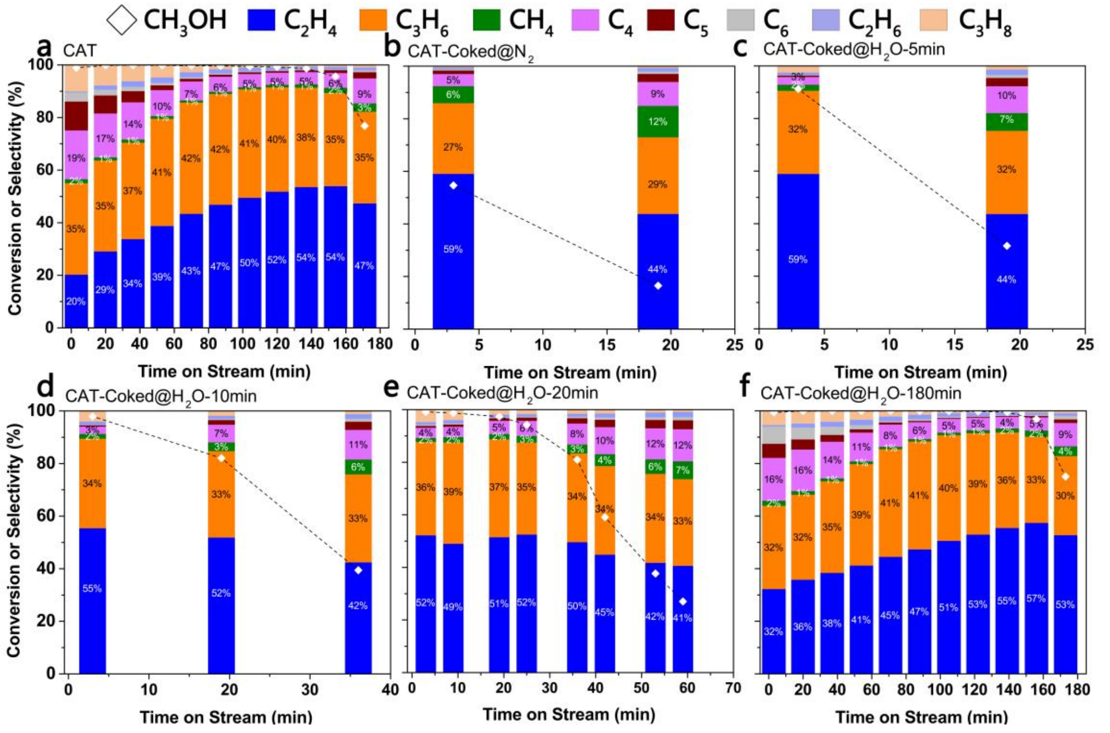

Supplementary Figure 8. MTO reaction catalyzed by (**a**) CAT catalyst, (**b**) CAT-Coked@N_2, (**c**) CAT-Coked@H_2O-5min, (**d**) CAT-Coked@H_2O-10min, (**e**) CAT-Coked@H_2O-20min and (**f**) CAT-Coked@H_2O-180min. MTO reaction was carried out at 723 K with 2.0 g_{MeOH} $g_{cat.}^{-1}$ h^{-1} and mass ratio of water to methanol in the feed of 0.2.

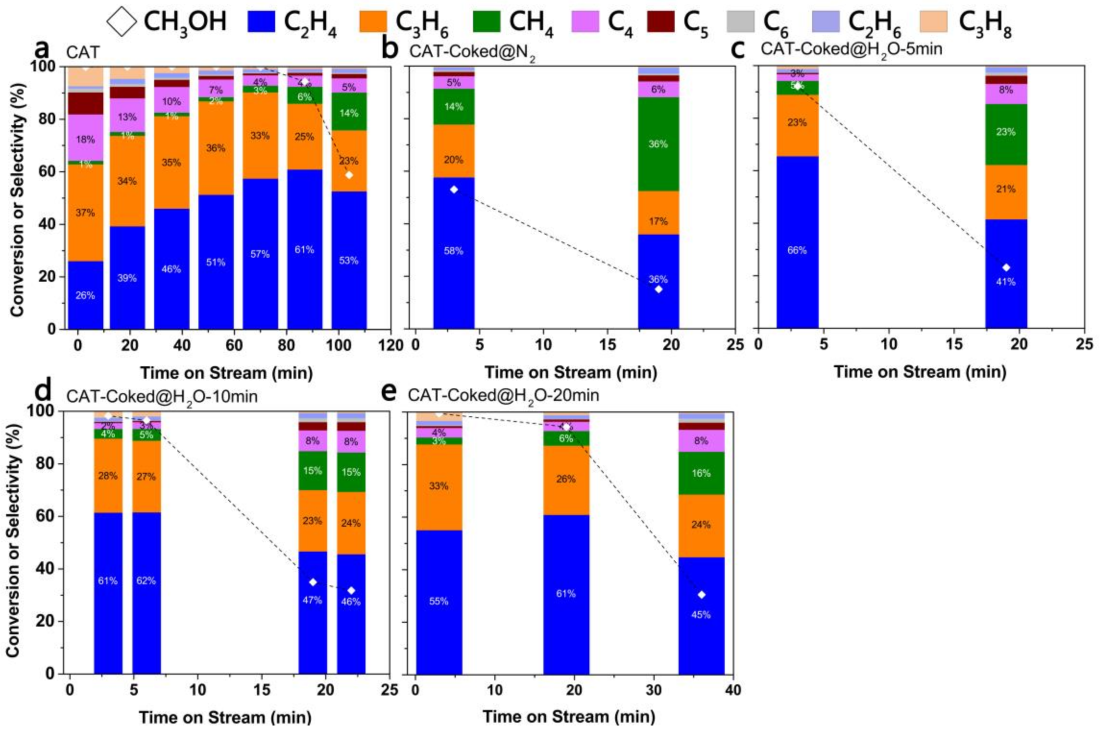

Supplementary Figure 9. MTO reaction catalyzed by (**a**) CAT catalyst, (**b**) CAT-Coked@N_2, (**c**) CAT-Coked@H_2O-5min, (**d**) CAT-Coked@H_2O-10min and (**e**) CAT-Coked@H_2O-20min. MTO reaction was carried out at 763 K with 2.0 g_{MeOH} $g_{cat.}^{-1}$ h^{-1} and mass ratio of water to methanol in the feed of 0.2.

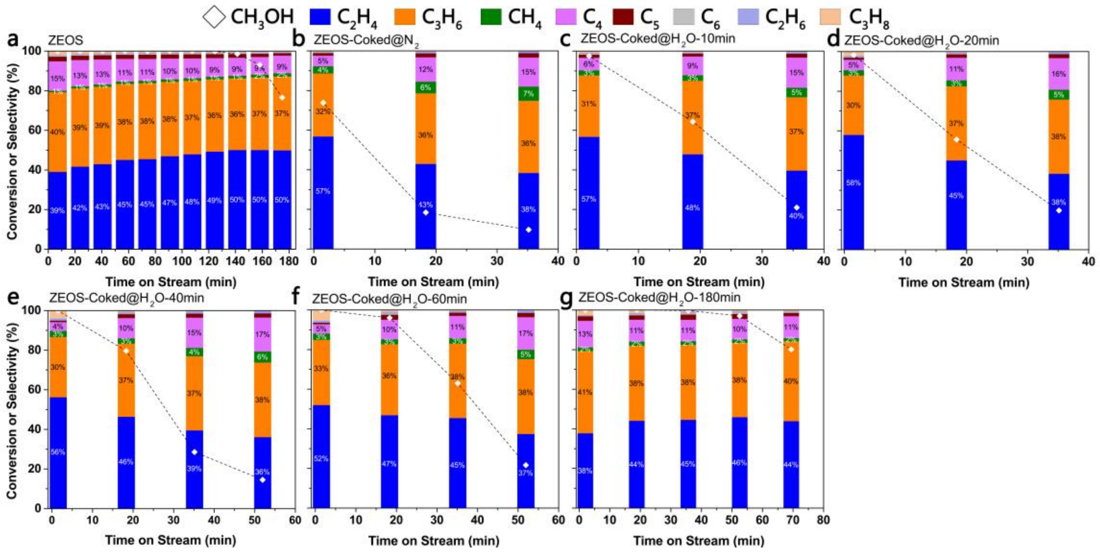

Supplementary Figure 10. MTO reaction catalyzed by (**a**) ZEOS, (**b**) ZEOS-Coked@N_2, (**c**) ZEOS-Coked@H_2O-10min, (**d**) ZEOS-Coked@H_2O-20min, (**e**) ZEOS-Coked@H_2O-40min, (**f**) ZEOS-Coked@H_2O-60min and (**g**) ZEOS-Coked@H_2O-180min. MTO reaction was carried out at 723 K with 6.6 g_{MeOH} $g_{cat.}^{-1}$ h^{-1} and mass ratio of water to methanol in the feed of 0.2.

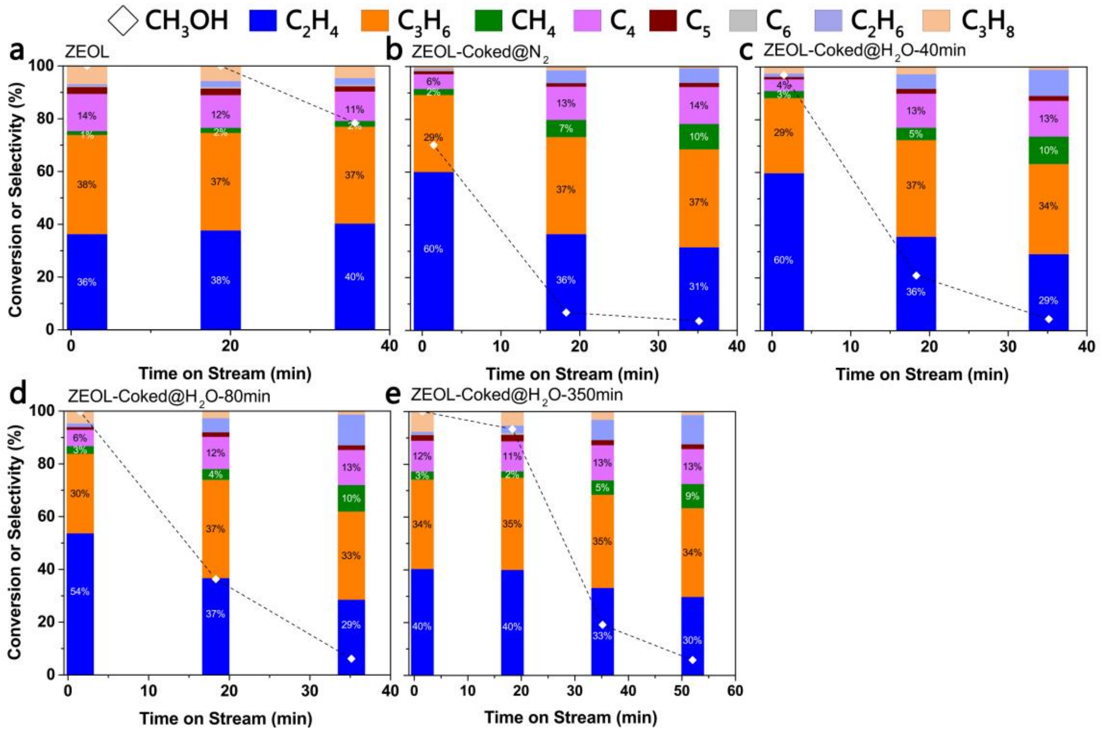

Supplementary Figure 11. MTO reaction catalyzed by (**a**) ZEOL, (**b**) ZEOL-Coked@N_2, (**c**) ZEOL-Coked@H_2O-40min, (**d**) ZEOL-Coked@H_2O-80min and (**e**) ZEOL-Coked@H_2O-350min. MTO reaction was carried out at 723 K with 6.6 g_{MeOH} $g_{cat.}^{-1}$ h^{-1} and mass ratio of water to methanol in the feed of 0.2.

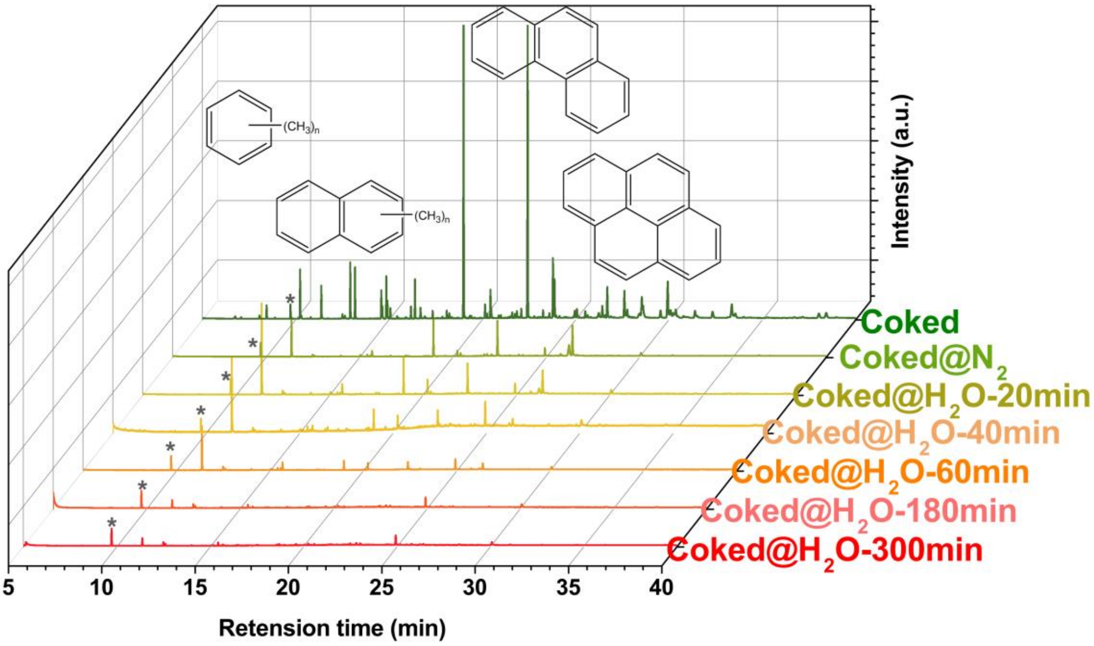

Supplementary Figure 12. GC-MS shows the detailed composition of coke species with molecular weight less than 200 Da in ZEOS-Coked and ZEOS-Coked treated by nitrogen or steam series samples. * represents the internal standard of hexachloroethane.

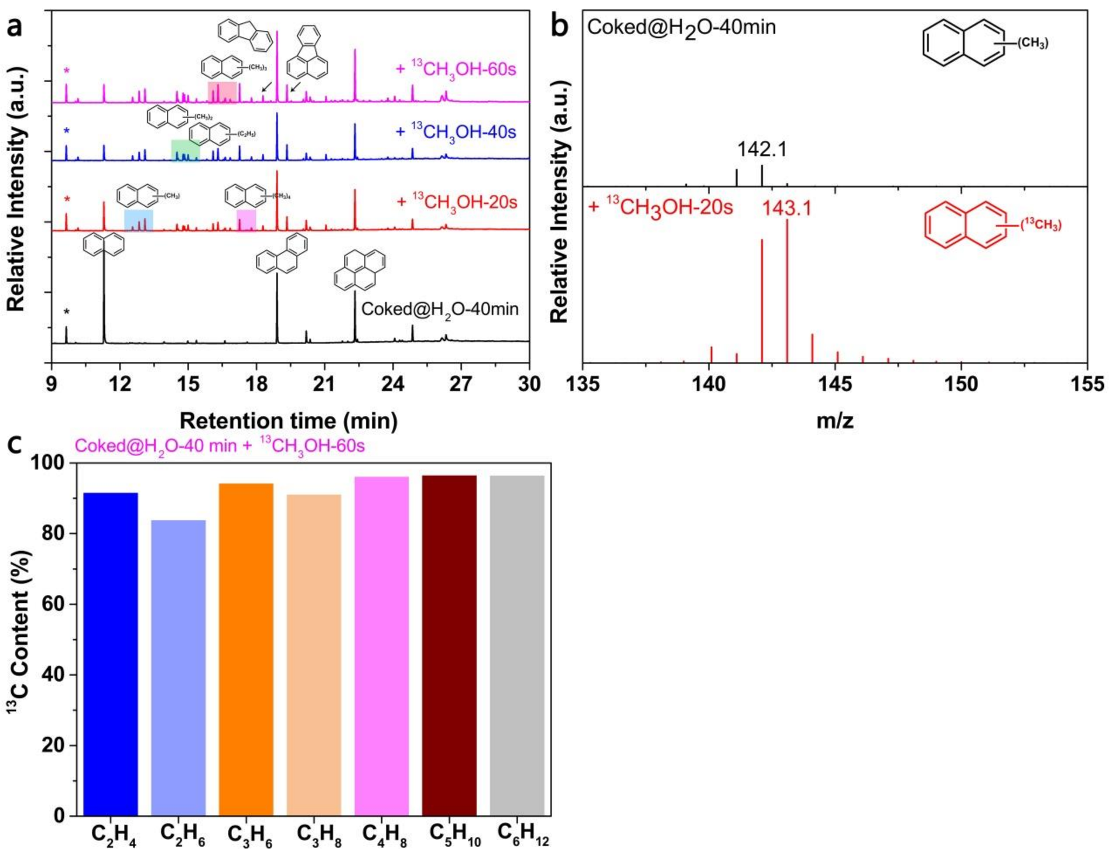

Supplementary Figure 13. (a) The detailed composition of coke species in ZEOS-Coked@H_2O-40min and quenched ZEOS-Coked@H_2O-40min reacted with ^{13}C-methanol at 20, 40 and 60 s analyzed by GC-MS. * represents the internal standard of hexachloroethane. (b) MS (retention time at 13.11 min in Supplementary Figure 13a) shows the incorporation of ^{13}C into naphthalene and formation of methyl-naphthalene with ^{13}C-methanol. (c) The gas product distribution over ZEOS-Coked@H_2O-40min sample reacted with ^{13}C-methanol for 60s.

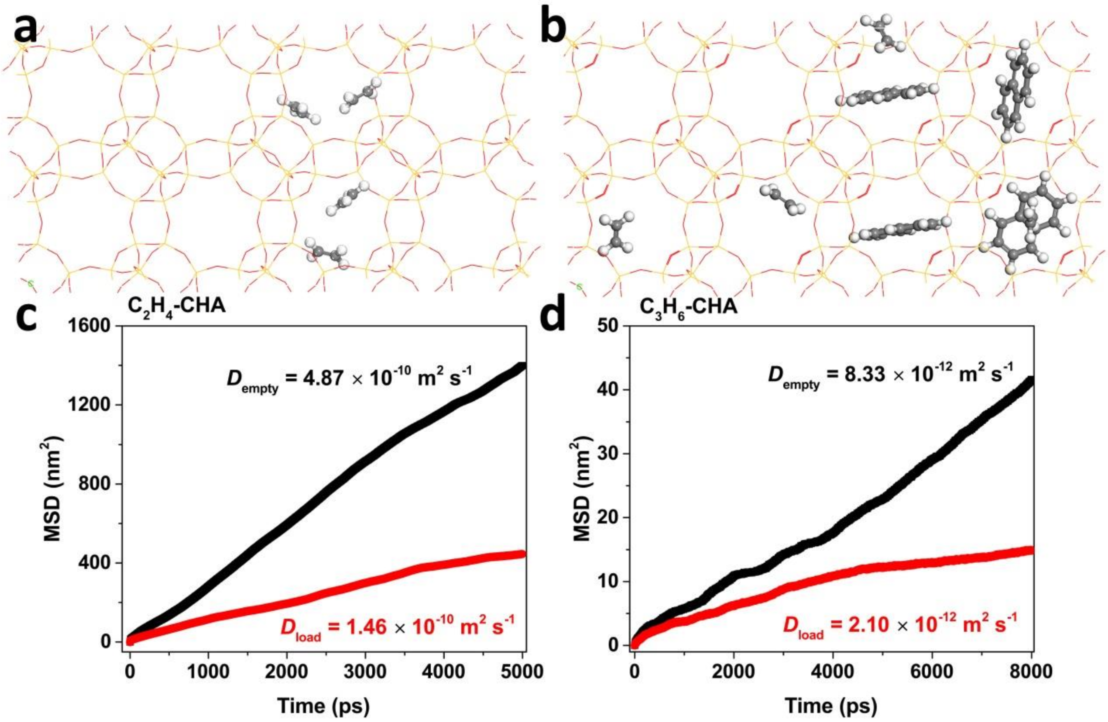

Supplementary Figure 14. (a, b) Structure of ethylene loaded (0.5 molecule per cage) in Si-CHA and Si-CHA loaded with 0.5 per cage naphthalene molecules. (c, d) MSD of ethylene and propylene diffusion in Si-CHA and Si-CHA loaded with naphthalene molecules at 723 K. Atom colors: C (grey), H (white), O (red) and Si (yellow).

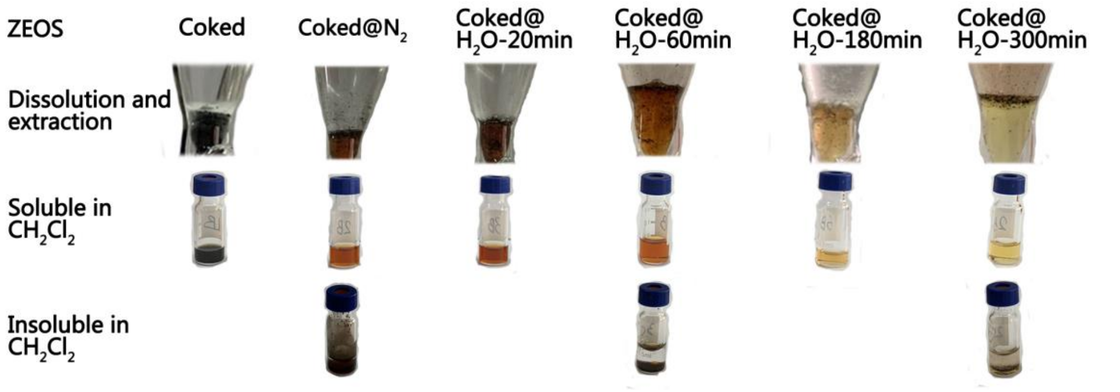

Supplementary Figure 15. Optical photographs of dissolution and extraction experiments of ZEOS-Coked, ZEOS-Coked@N_2, ZEOS-Coked@H_2O-20min, ZEOS-Coked@H_2O-60min, ZEOS-Coked@H_2O-180min and ZEOS-Coked@H_2O-300min samples.

As shown in Supplementary Figure 15, after treatment of ZEOS-Coked sample by nitrogen or steam at 953 K for different time, it can be visibly observed that black solids suspend above the extracted phase by dichloromethane, and these solids are defined insoluble carbonaceous species in dichloromethane.

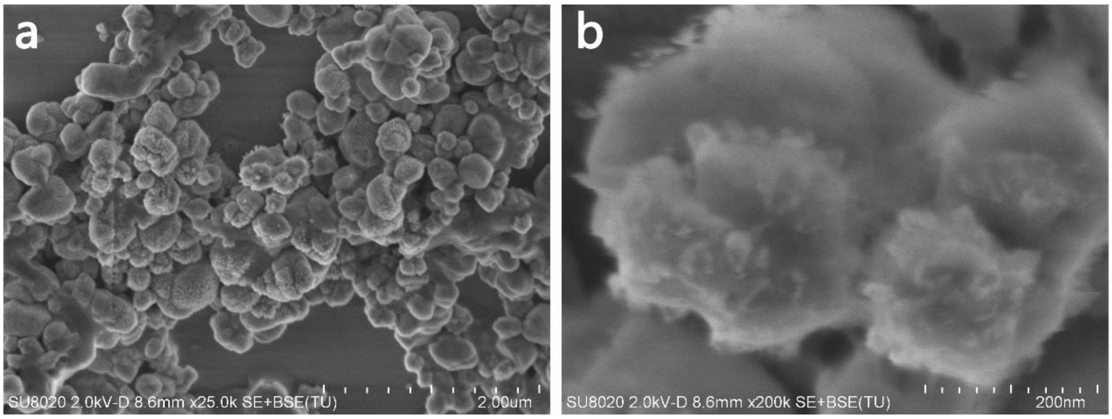

Supplementary Figure 16. (a) SEM image of the insoluble carbonaceous species in dichloromethane (Supplementary Figure 15). (b) Partially enlarged image of Supplementary Figure 16a.

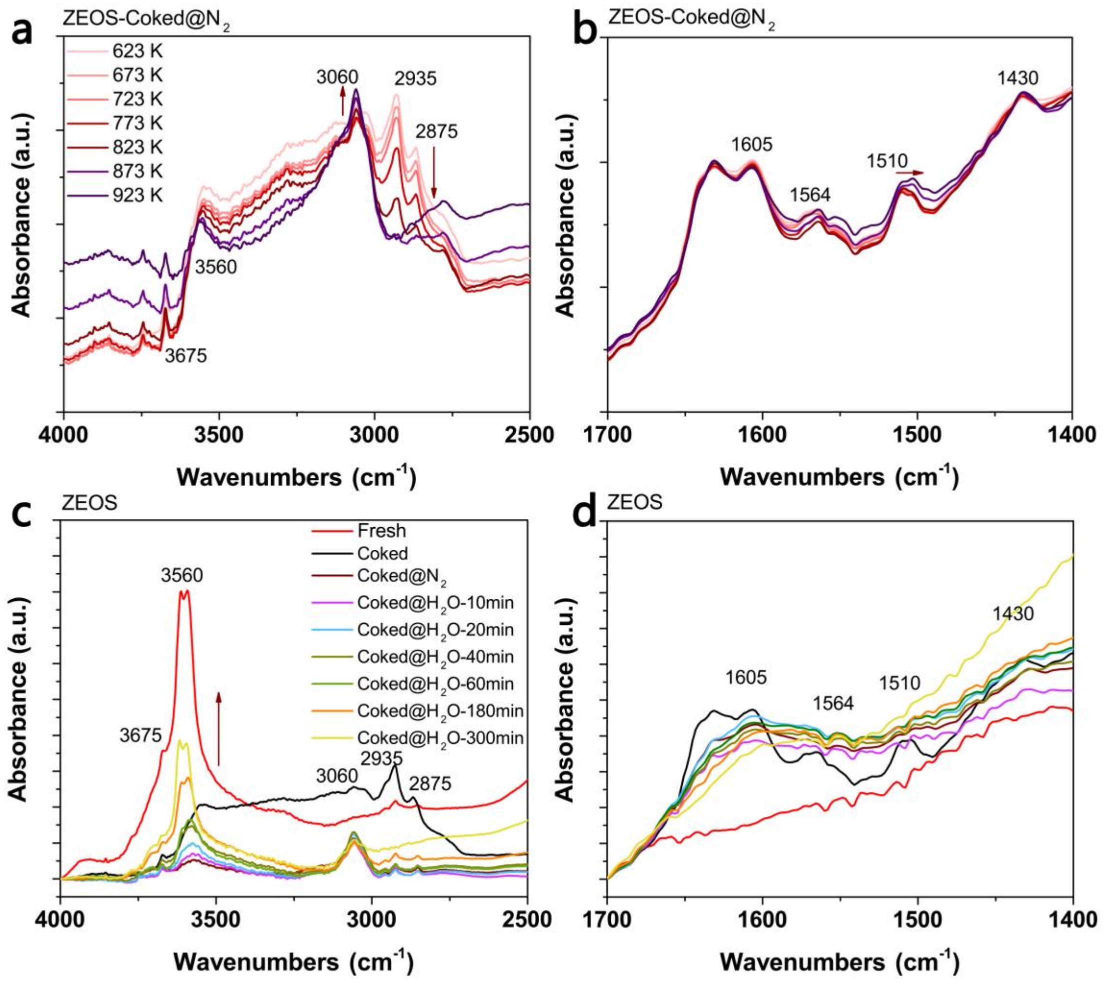

Supplementary Figure 17. DRIFT spectra of (**a, b**) ZEOS-Coked samples treated by nitrogen at high temperature of 723, 773, 823, 873, 923 and 973 K, respectively, and held for 20 min then cooled to 623 K to record spectra and (**c, d**) ZEOS-Coked samples treated by nitrogen and steam for different time at 953 K, which was compared with the spectra of fresh ZEOS and ZEOS-Coked. The ZEOS-Coked samples treated by steam at 953 K for 10, 20, 40, 60, 120 and 180 min in fixed-bed reactor.

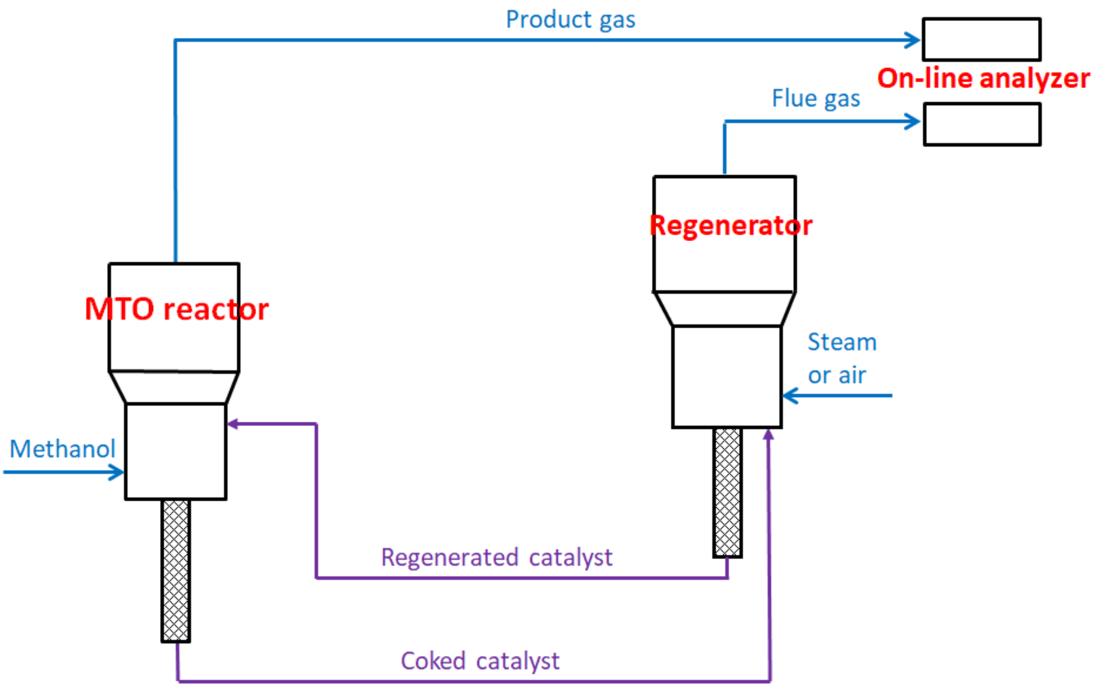

Supplementary Figure 18. Schematic diagram of the fluidized bed reactor-regenerator pilot plant used in this work.

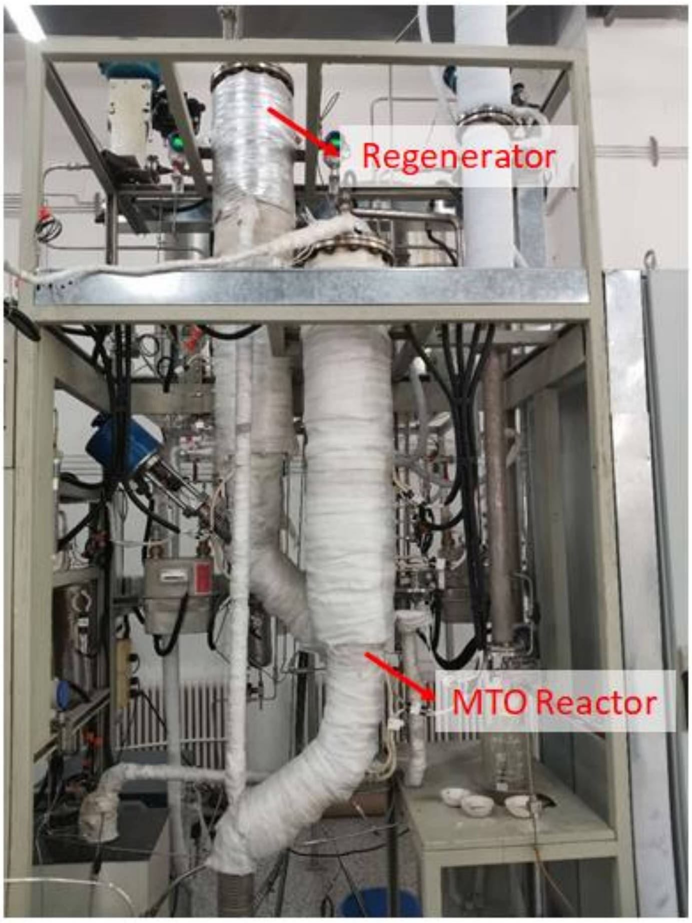

Supplementary Figure 19. Photo of the fluidized bed reactor-regenerator pilot plant used in this work.

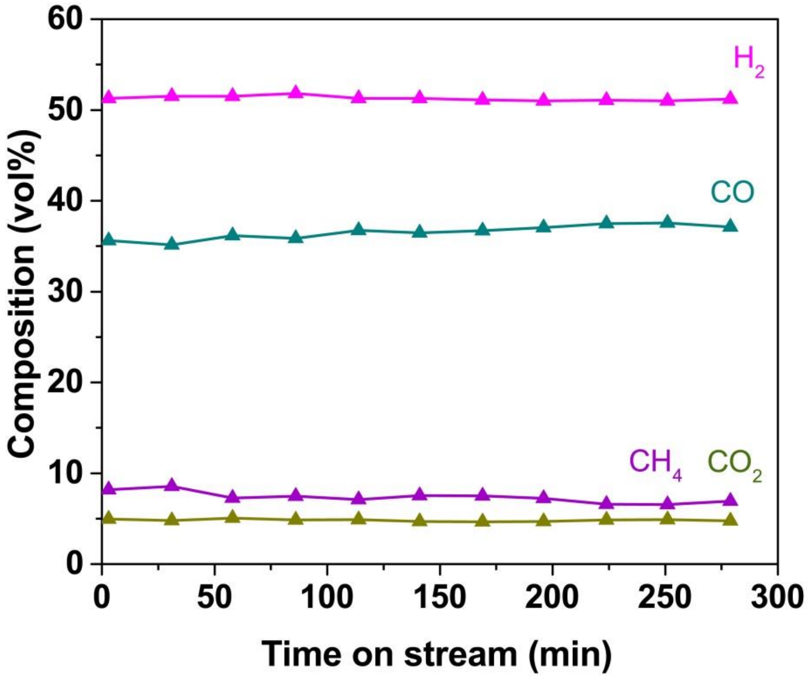

Supplementary Figure 20. Flue gas composition from regenerator during steam treatment of CAT-Coked sample at 953 K with the water feed of 300 g h^{-1} in the MTO reaction-regeneration circulating pilot facility.

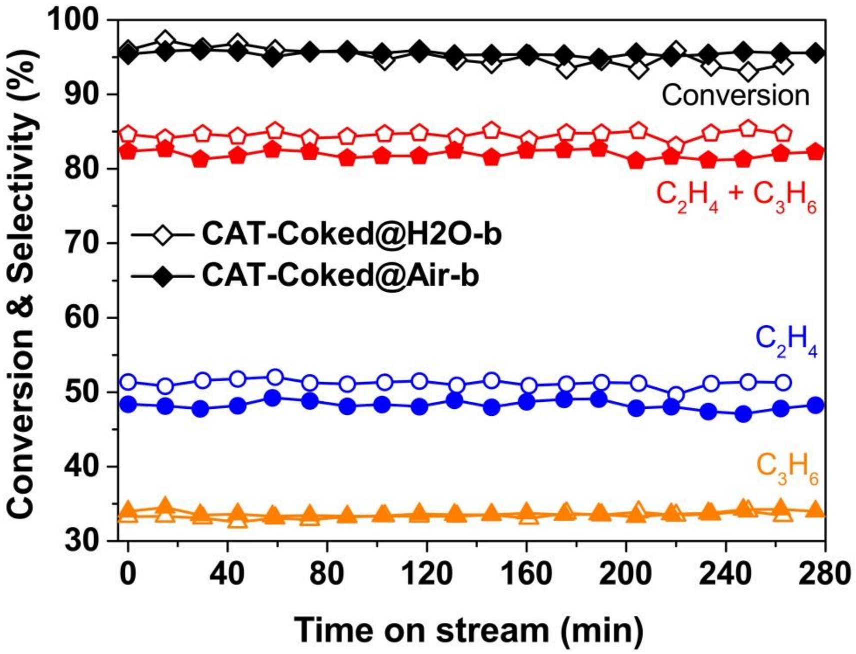

Supplementary Figure 21. MTO reaction performances over the CAT-Coked sample partially regenerated by air combustion at 953 K (abbreviated as CAT-Coked@Air-b) with the air feed of 30 L h^{-1} and treated by steam at 953 K with the water feed of 500 g h^{-1} (abbreviated as CAT-Coked@H_2O-b).

Supplementary Reference

1. Ferri, P. *et al.* Chemical and Structural Parameter Connecting Cavity Architecture, Confined Hydrocarbon Pool Species, and MTO Product Selectivity in Small-Pore Cage-Based Zeolites. *ACS Catal.*, 11542-11551 (2019).
2. Wragg, D. S. *et al.* SAPO-34 methanol-to-olefin catalysts under working conditions: A combined in situ powder X-ray diffraction, mass spectrometry and Raman study. *J. Catal.* **268**, 290-296 (2009).
3. Ferri, P. *et al.* Impact of Zeolite Framework Composition and Flexibility on Methanol-To-Olefins Selectivity: Confinement or Diffusion? *Angew. Chem. Int. Ed.* **59**, 19708-19715 (2020).
